# Prognosis and biological function of SGOL1 in clear cell renal cell carcinoma: a multiomics analysis

**DOI:** 10.1186/s12920-024-01825-7

**Published:** 2024-02-21

**Authors:** Zezhong Yang, Yunzhong Jiang, Lu Wang, Binghe Yu, Hui Cai, Jinhai Fan, Mengzhao Zhang

**Affiliations:** 1 Department of Urology, The First Affiliated Hospital of Xi’an Jiaotong University, Address: No.277 Yanta West Road, Xi’an, Shaanxi 710061 China; 2 Department of Ophthalmology, The First Affiliated Hospital of Xi’an Jiaotong University, Address: No.277 Yanta West Road, Xi’an, Shaanxi 710061 China; 3 Department of Vascular Surgery, The First Affiliated Hospital of Xi’an Jiaotong University. Address: No, 277 Yanta West Road, Xi’an, Shaanxi 710061 China

**Keywords:** SGOL1, ccRCC, Prognostic biomarkers, Immune infiltration, Malignant progression

## Abstract

**Background:**

Shugoshin-1 (SGOL1) is a mammalian ortholog of Shugoshin in yeast and is essential for precise chromosome segregation during mitosis and meiosis. Aberrant SGOL1 expression was reported to be closely correlated with the malignant progression of various tumors. However, the expression pattern and biological function of SGOL1 in clear cell renal cell carcinoma (ccRCC) are unclear.

**Methods:**

The Cancer Genome Atlas (TCGA) and Gene Expression Omnibus (GEO) databases provide mRNA expression data and outcome information for ccRCC patients. Immunohistochemistry (IHC) of ccRCC tissue chips verified SGOL1 protein expression in ccRCC patients. Data processing and visualization were performed with the UALCAN, TISIDB, TIMER, GSCA, LinkedOmics, and starBase databases. Gene Ontology (GO) annotation and gene set enrichment analysis (GSEA) were used to identify SGOL1-related biological functions and signaling pathways. Immune infiltration analysis was performed using the TISIDB database, ssGSEA algorithm, and TCGA-KIRC cohort. The biological role of SGOL1 in ccRCC was investigated using a series of in vitro cytological assays, including the MTT assay, EdU staining assay, flow cytometry analysis, Transwell assay, and wound healing assay.

**Results:**

SGOL1 was highly expressed in ccRCC and linked to adverse clinicopathological parameters and unfavorable prognosis. Multivariate logistic regression and nomogram calibration suggested that SGOL1 might serve as an independent and reliable prognostic predictor of ccRCC. Functional enrichment analysis indicated that SGOL1 may be involved in the cell cycle, the p53 pathway, DNA replication, and T-cell activation. Furthermore, tumor microenvironment (TME) analysis suggested that SGOL1 was positively associated with Treg infiltration and immune checkpoint upregulation. In addition, we identified a potential SNHG17/PVT1/ZMIZ1-AS1-miR-23b-3p-SGOL1 axis correlated with ccRCC carcinogenesis and progression. Finally, we demonstrated that SGOL1 promoted ccRCC cell proliferation, migratory capacity, and invasion in vitro.

**Conclusions:**

SGOL1 potentially functions as an oncogene in ccRCC progression and might contribute to the immunosuppressive TME by increasing Treg infiltration and checkpoint expression, suggesting that targeting SGOL1 could be a novel therapeutic strategy for the treatment of ccRCC patients.

**Supplementary Information:**

The online version contains supplementary material available at 10.1186/s12920-024-01825-7.

## Introduction

Renal cell carcinoma (RCC), a kidney parenchyma cancer, is the most common (~ 90%) and lethal subtype of kidney cancer. As the most common pathological type of RCC, ccRCC accounts for approximately 85% of new patients and is characterized mainly by the loss of the tumor suppressor Von Hippel Lindau (VHL) function, the mutations in the factors governing the hypoxia signaling pathway and intracellular lipid accumulation caused by unknown pathomechanisms [1, 2]. Due to the insensitivity of ccRCC to adjuvant chemotherapy and radiotherapy, surgery is still the dominant treatment method for this disease, but postoperative recurrence and metastasis are common [3]. At present, great progress has been made in the research of targeted gene regulation in the progression and prognosis of kidney disease [4]. Meanwhile, small molecule agents that target VEGF or mTOR, such as bevacizumab and everolimus, have improved the survival of ccRCC patients to a certain extent. However, a considerable number of patients still have a poor prognosis due to drug resistance [5–7]. Currently, immunotherapy-based combination treatments have demonstrated therapeutic efficacy in ccRCC patients, especially those with advanced ccRCC. Additionally, immunotherapy based on immune checkpoint inhibitors (ICIs) has been approved for the treatment of ccRCC, which is a crucial step in expanding the treatment options for ccRCC patients and has significantly improved the clinical outcomes of patients with ccRCC [8, 9]. As a heterogeneous disease with severe immune cell infiltration, discovering novel and reliable immune-related biomarkers and therapeutic targets for ccRCC would be tremendously useful for improving the clinical prognosis and immunotherapy efficacy of ccRCC patients.

Numerous studies have emphasized the role of genetic instability as a causative agent in the development of abnormal chromosome segregation in humans, which can ultimately lead to the formation of tumors [10]. Previous extensive studies have identified and confirmed the Shugoshin family (SGOL1/2), as an important protector for centromeric cohesion to maintain the stability of the chromosome and ensure proper genome separation, is extremely important in the meiosis of eukaryotic cells [11]. Available data suggest that SGOL1 exclusively handles many functions of SGOL2 and its splice variants in eukaryotes, and SGOL1, as the principal isoform of the Shugoshin family, has attracted more and more attention [12]. Shugoshin-1 (SGOL1) is a human ortholog of the Shugoshin in yeast located on Chromosome 3p24.3 that exerts a vital role in maintaining chromosome cohesion and preventing premature chromosome segregation and chromosomal instability (CIN) [13–15]. Given the substantial involvement of mitosis in tumorigenesis, cancer progression, and cancer treatment [16], the potential role of SGOL1 in cancer is worth further research. Current studies have identified that SGOL1 is upregulated and serves as a prognostic and therapy-related biomarker in various cancers, such as breast cancer, hematological malignancies, lung cancer, glioma, colon cancer, and hepatocellular carcinoma [17–20]. In addition, SGOL1 can affect the malignant progression of tumors by altering their biological function. For instance, high expression of SGOL1 can promote the proliferation and metastasis of prostate cancer through an AKT-dependent pathway and increase the multidrug resistance of gastric cancer cells [21, 22]. The oncogenic role of SGOL1 is well-recognized in various cancers. However, to date, the impact of SGOL1 on the prognosis of patients with ccRCC and the efficacy of immunotherapy is still poorly understood.

In this study, we identified the expression pattern of SGOL1 and evaluated its correlation with the clinical features and outcomes of patients with ccRCC using multiple databases, including the TCGA and GEO databases. The potential biofunctions and signaling pathways associated with SGOL1 were subsequently investigated through Gene Ontology (GO) and Kyoto Encyclopedia of Genes and Genomes (KEGG) analyses and gene set enrichment analysis (GSEA). Afterward, we analyzed the correlation between SGOL1 expression and the proportions of tumor-infiltrating immune cells (TIICs) and immune checkpoint inhibitors. Next, we identified a potential lncRNA–miRNA-mRNA regulatory network associated with ccRCC carcinogenesis and progression. Finally, the cellular biological function of SGOL1 in ccRCC was investigated using a series of in vitro cytological assays. Our findings may help to deepen the understanding of the pathophysiological process of ccRCC and provide a reliable prognostic biomarker and a novel immune-associated therapeutic target for ccRCC.

## Materials and methods

### Data acquisition and preprocessing

A transcriptomic dataset of ccRCC patients with corresponding clinical information, including age, gender, cancer subtype, nodal metastasis status, pathological stage, TNM stage, histological grade, and outcome details, was obtained from The Cancer Genome Atlas Kidney Renal Clear Cell Carcinoma (TCGA-KIRC) database [23](https://portal.gdc.cancer.gov/). The TCGA-KIRC dataset contained 541 ccRCC tumor tissues and 72 peritumoral normal tissues. Two GEO datasets (GSE16449 and GSE40435) were obtained from the NCBI-GEO database [24, 25]; GSE40435 consisted of 101 ccRCC tissues and matched peritumoral normal tissues [26], and GSE16449 consisted of 52 ccRCC tissues and 18 normal kidney tissues [27] (https://www.ncbi.nlm.nih.gov/geo/). All the data and clinical information were normalized with the limma R package and analyzed and visualized with R (4.2.1) software.

### mRNA expression analysis

Data on SGOL1 expression in various cancer types were obtained from the TIMER database [28]. To determine the differential expression of SGOL1 in ccRCC, the ggplot2 (V3.4.0) package was used to evaluate the RNA-seq data from the TCGA and GEO databases. The Wilcoxon rank-sum test was used to assess the statistical significance of differences in SGOL1 expression between different groups. The difference in SGOL1 expression between normal kidney tissues and ccRCC tissues was examined using the Weltch t-test. Furthermore, the UALCAN database [29] was utilized to investigate the associations between SGOL1 mRNA expression and diverse clinical characteristics, including age, gender, ccRCC subtype, lymph node metastasis, clinical stage, and tumor grade. P < 0.05 was considered to indicate statistical significance.

### Prognostic and diagnostic value analysis

The receiver operating characteristic (ROC) curve used to evaluate the predictive accuracy of SGOL1 in ccRCC was constructed by the R package pROC [30]. The statistical analysis of clinical outcomes, such as overall survival (OS), disease-specific survival (DSS), and progression-free survival (PFI), was performed by the survminer (V0.4.9) package surv_cutpoint function and visualized by ggplot2 based on the TCGA-KIRC cohort, in which patients were classified into high-SGOL1-expression and low-SGOL1-expression groups [31]. The correlation of patient OS with different clinical variables (age, race, gender, TNM stage, pathologic stage, and histologic grade) was examined using Kaplan–Meier curves and the log-rank test based on SGOL1 expression. We also conducted univariate and multivariate Cox regression analyses to evaluate the effect of SGOL1 expression on clinicopathological features, including age, TNM stage, pathological stage, histological grade, and survival, in ccRCC patients [32]. Then, we created a nomogram to predict 1-, 3-, and 5-year survival probabilities based on the predictors provided by incorporating the above independent prognostic factors. p < 0.05 was considered to indicate statistical significance.

### Coexpression network and functional enrichment analysis

Using the LinkedOmics database [33], a coexpression gene network was constructed, and differentially expressed genes (DEGs) between the high-SGOL1-expression group and low-SGOL1-expression group in the TCGA-KIRC cohort were identified and are illustrated in the form of a volcano map and heatmap. Spearman’s correlation analysis was performed, and p < 0.05 was considered significant. Functional enrichment analysis, including GO annotation and KEGG analysis, was performed via the clusterProfiler (V4.4.4) package based on the top 1000 DEGs correlated with SGOL1. The ggplot2 package was used to visualize the GO annotation and KEGG results. A value of *P* < 0.05 and a false discovery rate (FDR) < 0.05 were considered significant.

### Immune infiltration and immune checkpoint analysis

Based on the TISIDB database [34], the correlation between SGOL1 expression and enrichment of 28 tumor-infiltrating lymphocytes (TILs) in various tumors was confirmed. Using the TCGA database (https://portal.gdc.cancer.gov), we downloaded the TCGA-KIRC SGOL1 RNAseq data. Based on the ssGSEA algorithm provided in the R package -GSVA [35] and the expression data of SGOL1 extracted from the TCGA-KIRC cohort, the expression data of SGOL1 and the markers of 24 immune cells were calculated to determine the correlation between the expression of SGOL1 and the infiltration enrichment of 24 immune cells. Then, we examined the relationship between SGOL1 expression and immune checkpoint inhibitor (ICI) expression using the Sangerbox database, which is a comprehensive, user-friendly platform for bioinformatics analysis and provides customizable analysis tools, including various bioinformatics online analysis tools, correlation analysis of immune cell infiltration and immune checkpoint expression, clinical prognosis analysis and gene mutation expression analysis [36]. The associations between SGOL1 and immune checkpoint inhibitors extracted from the Sangerbox database were investigated and visualized as scatter plots. The clinical outcomes of overall survival (OS) in ccRCC patients with high and low SGOL1 expression and diverse immune cell infiltration enrichment (NK cells and Tregs) were investigated via the TIMER 2.0 website [37].

### LncRNA–miRNA–mRNA network prediction

The potential upstream miRNAs targeting SGOL1 mRNA were selected and predicted by the Gene Set Cancer Analysis (GSCA) database, a comprehensive database consisting of verified databases (papers, StarBase, miRTarBase, and mir2disease) and prediction databases (TargetScan and miRanda) [38]. The miRNA and SGOL1 mRNA expression data were extracted from the TCGA-KIRC dataset, and correlation analysis was subsequently performed; the results are presented as a networkD3 plot. The FDR-adjusted P value was used, and genes with FDR <  = 0.05 and R < 0 were preserved. The potential lncRNAs targeting candidate miRNAs were selected and subjected to correlation analysis with SGOL1 expression based on the TCGA-KIRC cohort, after which the lncRNAs positively associated with SGOL1 were retained. The StarBase and TargetScan databases further confirmed the relationship and correlation of the potential lncRNAs with SGOL1 expression [39, 40]. Spearman correlation analysis was used to assess the relationships between lncRNAs, miRNAs, and SGOL1 expression. Survival analysis of the TCGA-KIRC cohort correlated with potential lncRNAs and miRNAs was performed with the survminer package, and the results were visualized with the ggplot2 package in R (4.2.1) software. p < 0.05 was considered to indicate statistical significance.

### ccRCC tissue chips and immunohistochemistry (IHC) staining

Outdo Biotech Co., Ltd. (Shanghai, China) generated ccRCC tissue chips (catalog no. HKid-CRCC060PG-01) containing 30 individual ccRCC patient tissues and corresponding normal tissues. The differential expression of SGOL1 between ccRCC tissues and peritumoral normal tissues was validated by an immunohistochemical staining assay, the procedure for which was described in detail in our previous article [41]. Immunostaining was performed with an SGOL1 polyclonal antibody (Proteintech, Cat No: 16977–1-AP).

### Cell culture and cell transfection

The ccRCC cell lines SW839, RCC-4, 769-P, A498, Caki-1, 786-O, OS-RC-2, and HK-2, as well as the human renal proximal tubule cell line HK-2, were purchased from the American Type Culture Collection (ATCC, USA) and cultured in RPMI-1640 containing 10% FBS. All cell lines were incubated at 37°C in a humidified atmosphere of 5% CO2. Double-stranded siRNA oligonucleotides against SGOL1 were designed and synthesized by GenePharma (Shanghai GenePharma Co.) to downregulate SGOL1 expression in 786-O cells. For SGOL1 inhibition, the selected targeting sequences were as follows: si-NC: 5’-UUCUCCGAACGUGUCACGUTT-3’; si-SGOL1: 5’-AUAGCUGCACCAUGCCAAAUATT-3’. Moreover, the SGOL1 overexpression plasmid (SGOL1) and empty vector plasmid (control) were synthesized and obtained from GeneCopoeia Company (Guangzhou, China) based on the pcDNA 3.1 vector and transfected into SW839 cells with Lipofectamine 3000 reagent according to the manufacturer’s protocols (Invitrogen, Carlsbad, CA, USA).

### Western blotting

The proteins were extracted from ccRCC cells and HK-2 cells with RIPA buffer (Beyotime Institute of Biotechnology, China) supplemented with 1% protease and phosphatase. The total protein concentration was subsequently detected with a BCA kit (Thermo, Germany). The proteins (20 μg) were subjected to SDS–PAGE and transferred onto polyvinylidene fluoride membranes (FVDF, 0.45 μm; Merck Millipore). After being blocked with 5% skim milk for 1 h and incubated with anti-SGOL1 (Proteintech, 16,977–1-AP), anti-P21 (Proteintech, 10,355–1-AP), anti-cyclin D1 (Proteintech, 26,939–1-AP), anti-CDK2 (Proteintech, 10,122–1-AP), anti-β-actin (Proteintech, 20,536–1-AP), anti-MMP2 (CST, 40,994), anti-MMP9 (CST, 13,667), anti-cyclin E1 (CST, 20,808), anti-E-cadherin (CST, 3195), and anti-N-cadherin (CST, 13,116) antibodies at 4°C overnight, the PVDF bands were incubated with secondary antibodies and detected by enhanced ECL chemiluminescent assay and visualized by Bio-Rad software after washing with TBST three times.

### Cell proliferation assay

Briefly, 96-well plates containing transfected cells (786-O and SW839) were cultured for 24, 48, 72, or 96 h in the cell incubator, after which CCK-8 reagent was added to each well of the 96-well plates. The absorbance at 450 nm was detected by an EnSpire Multimode Plate Reader (PerkinElmer) after the cells were incubated with the CCK-8 reagent for 1 h. Changes in cell viability are presented as line graphs. Triplicate experiments were performed for each assay.

### Wound healing assay

A 100 μl plastic pipette tip was used to scratch the cell monolayer after the transfected cells reached 100% confluence in a 6-well plate. After being washed with PBS, the cells in the wound were incubated in a serum-free medium for a certain time (24 h). Finally, the wounds were photographed using a microscope (Olympus, Tokyo, Japan).

### Transwell migration and invasion assays

Then, 800 μl of RPMI 1640 medium supplemented with 10% FBS was added to the lower chamber, and 200 μl of RPMI 1640 serum-free medium supplemented with 3 × 10 [4] cells was added to the upper chamber (Corning, NY, USA). For cell migration, the cells were incubated in Boyden chambers (Millipore, Germany) for 24 h, fixed with 4% paraformaldehyde, stained with 0.5% crystal violet, and then imaged and counted with an inverted light microscope at × 100 magnification at five random fields. Following the same procedures, the upper chamber precoated with Matrigel (BD Biosciences, San Diego, CA, USA) was used to assess cell invasion.

### EdU assay

EdU labeled with Alexa Fluor 594 (Beyotime, China) was used to detect the DNA replication capacity of the transfected cells. The cells were cultured in a complete medium containing 10 μM EdU for 2 h in a cell incubator after washing with PBS 3 times. Then, the cells were fixed with 4% paraformaldehyde for 10 min and stained with DAPI for 5 min for nuclear staining. Finally, images of five random fields were captured at 100 × magnification under an Olympus fluorescence microscope (Tokyo, Japan). The intensity of the EdU staining was analyzed and calculated by ImageJ software and is presented as the fold change compared with the control.

### Cell flow cytometry assay

CcRCC cells (786-O and SW839) were collected from 6-cm dishes and resuspended in PBS under specific treatments. Then, 70% ice-cold ethanol was used to fix the cells at 4°C overnight. After the cells were suspended in PBS supplemented with 50 μg/ml propidium iodide (PI) and 100 μg/ml RNase A (1,1) for 15 min in the dark, the staining signals were evaluated using a FACSCalibur (Becton Dickinson), and the distribution of cells in each cell cycle phase was analyzed by Cell Quest software version 3.3 (BD Biosciences) and presented in the form of bar graphs.

### Luciferase reporter assay

Cells at 70% confluency were cotransfected with SGOL1 plasmids (wt-SGOL1, mut-SGOL1), which contained the binding sites of miR-23b-3p (GenePharma, Shanghai, China), and miR-23b-3p mimics or mimics control with Lipofectamine 3000 reagent (Invitrogen, Carlsbad, CA) according to the manufacturer’s protocols. The luciferase activity in each group was determined by a Tecan Infinite M200 plate reader with the Promega Dual-Luciferase® Reporter Assay System (Madison, WI, USA). *Renilla* luciferase activity was used as an internal control.

### Immunofluorescence microscopy

The spatial distribution of SGOL1 proteins in cells is available in version 7.0 of the Human Protein Atlas (HPA) database [42](http://www.proteinatlas.org). HPA is a comprehensive human protein expression analysis database containing 11,200 unique protein expression profiles and a map of protein expression patterns in normal and cancer cells and tissues. The HPA database offers a vital source of information for molecular biology research, including biomarker discovery efforts, identification of protein cellular locations and spatial distribution of proteins.

### Statistical analysis

R software version 4.2.1 and GraphPad Prism version 9.0 were used to analyze and visualize the results of the present study. Student’s t-tests or one-way ANOVA were used to compare the differences between groups. Spearman analysis was used to compute the correlation coefficients in this study. Unless otherwise stated, the data are shown as the mean ± SD of three independent experiments. *P* < 0.05 was considered to indicate statistical significance, and *P* < 0.01 was considered to indicate statistical significance.

## Results

### SGOL1 was upregulated in ccRCC

**Fig. 1:**
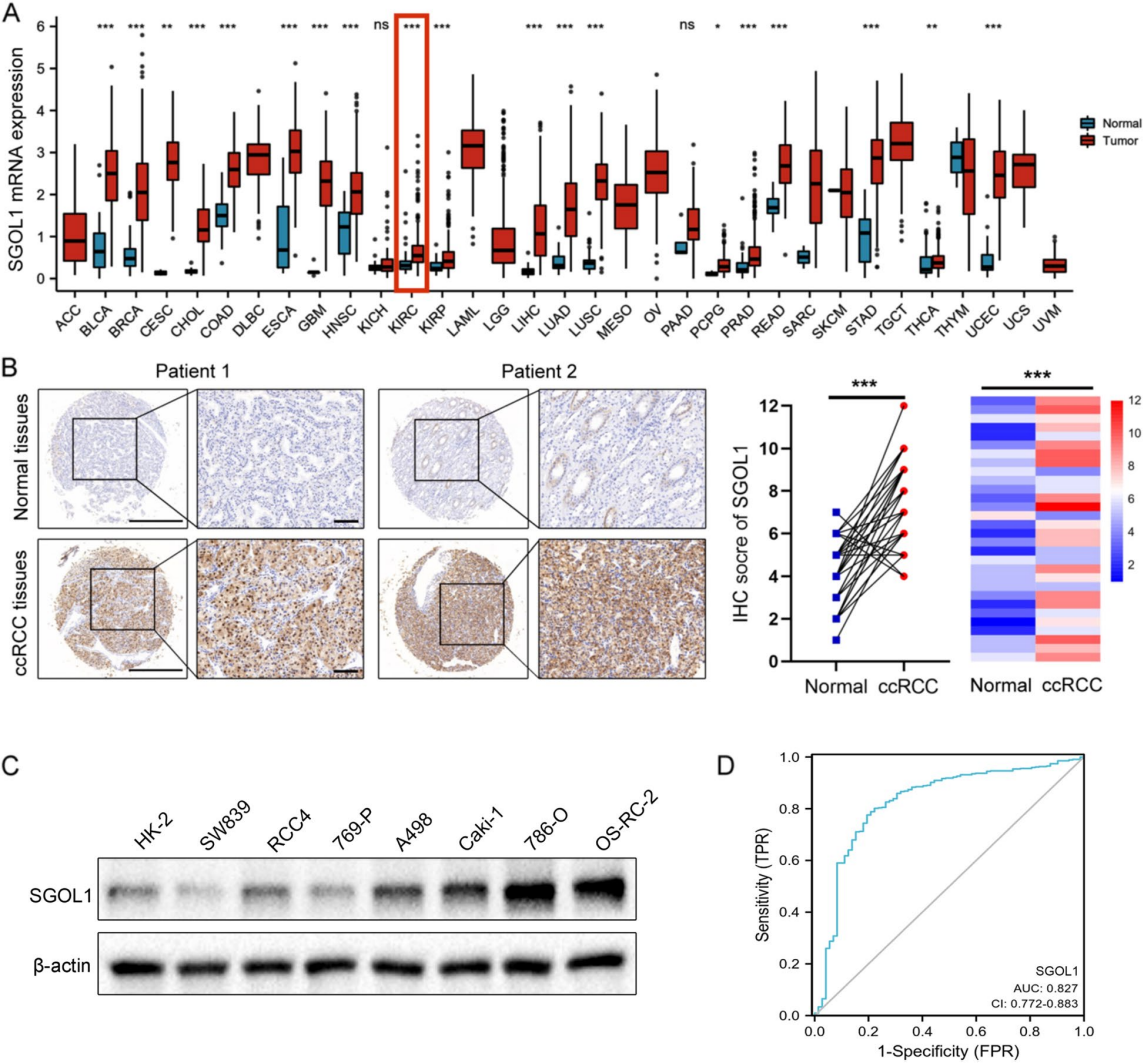
The expression of SGOL1 is upregulated in ccRCC. **A** SGOL1 mRNA expression according to a pan-cancer analysis. **B** Representative IHC images of SGOL1 in ccRCC tissues and matched peritumoral normal tissues; *n* = 30. The IHC staining score of SGOL1 was analyzed and presented as scatter plots and heatmaps. **C** The protein expression of SGOL1 in different ccRCC cell lines and HK-2 cells. **D** Receiver operating characteristic (ROC) curve of SGOL1 expression in the TCGA-KIRC cohort. (*P < 0.05, **P < 0.01, ***P < 0.001). The red line indicates the difference in the expression of SGOL1 between ccRCC tissues and normal tissues

The TCGA pan-cancer analysis indicated that SGOL1 mRNA was highly upregulated in almost all cancers, including ccRCC (Fig. 1A). We then extracted the mRNA expression profile of SGOL1 from the TCGA-KIRC and GEO datasets (GSE16449 and GSE40435) to further analyze SGOL1 mRNA expression in ccRCC. The analysis confirmed that SGOL1 mRNA expression was upregulated in ccRCC (Supplementary Fig. 1A). IHC analysis of samples from the ccRCC tissue chip, which included 30 pairs of ccRCC tissues and matched peritumoral normal tissues, indicated that SGOL1 was highly expressed in ccRCC tissues compared with normal tissues (Fig. 1B), and a similar result was also observed in ccRCC cell lines (Fig. 1C, supplementary Fig. 2). Using a ROC curve, the effectiveness of SGOL1 in differentiating between normal tissues (*n* = 72) and ccRCC tissues (*n* = 541) was assessed. The area under the curve (AUC) of SGOL1 was 0.827 (CI = 0.772–0. 883), which showed that SGOL1 had a high predictive value in ccRCC and could serve as a reliable diagnostic biomarker (Fig. 1D). Overall, these results suggest that SGOL1 is highly expressed in ccRCC.

### Correlations between SGOL1 Expression and diverse clinical characteristics

**Fig. 2:**
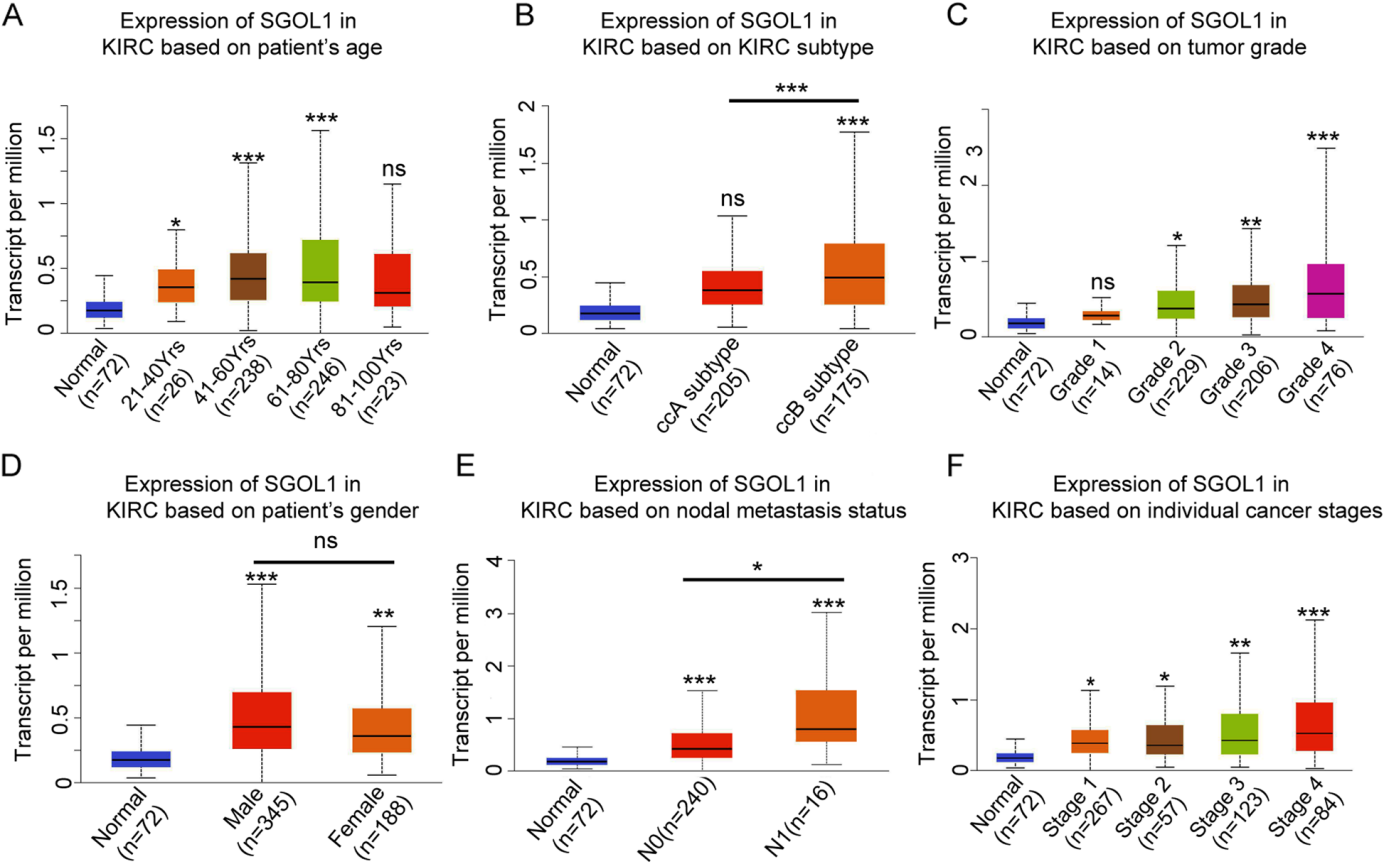
Correlation analysis between SGOL1 expression and diverse clinicopathological characteristics (**A**) The expression of SGOL1 increased with age. **B** Patients in the ccB subtype had higher SGOL1 expression than those in the ccA subtype. **C** SGOL1 expression was positively correlated with tumor grade in ccRCC patients. **D** SGOL1 expression did not differ between genders. **E** SGOL1 expression was positively correlated with lymph node metastasis. (F) SGOL1 expression was positively correlated with cancer stage in ccRCC patients. (*P < 0.05, ** P < 0.01, *** P < 0.001)

The relationship between differential clinicopathological characteristics and SGOL1 mRNA expression in the TCGA-KIRC cohort was determined via the UALCAN database. We found that high SGOL1 mRNA expression was significantly correlated with adverse clinical pathological parameters, including KIRC subtype, tumor grade, cancer stage, and nodal metastasis status (Fig. 2 B, C, E, F). The results also suggested that the mRNA level of SGOL1 increased with age but was not significantly related to age > 80 years (Fig. 2A). Moreover, the mRNA level of SGOL1 was not significantly different between male and female patients (Fig. 2D). According to Brannon's study, renal cancer can be divided into ccA and ccB types based on their gene expression profile and prognostic information, and patients with the ccB type usually exhibit worse overall survival (OS) [26]. According to UALCAN, SGOL1 expression was greater in patients with the ccB subtype than in those with the ccA subtype, which was consistent with our prediction. Moreover, upregulated SGOL1 expression was correlated with increased lymph node metastasis, advanced clinical stage, and increased tumor grade. All of the outcomes are displayed in Supplementary Table 1. Supplementary Table 2 shows similar results obtained from the TCGA-KIRC dataset cohort. All the data above indicated that SGOL1 overexpression was associated with adverse clinicopathological characteristics and may impact tumor initiation and progression, which indicated that SGOL1 could be a diagnostic biomarker for ccRCC.

### Prognostic value of SGOL1 in ccRCC

**Fig. 3:**
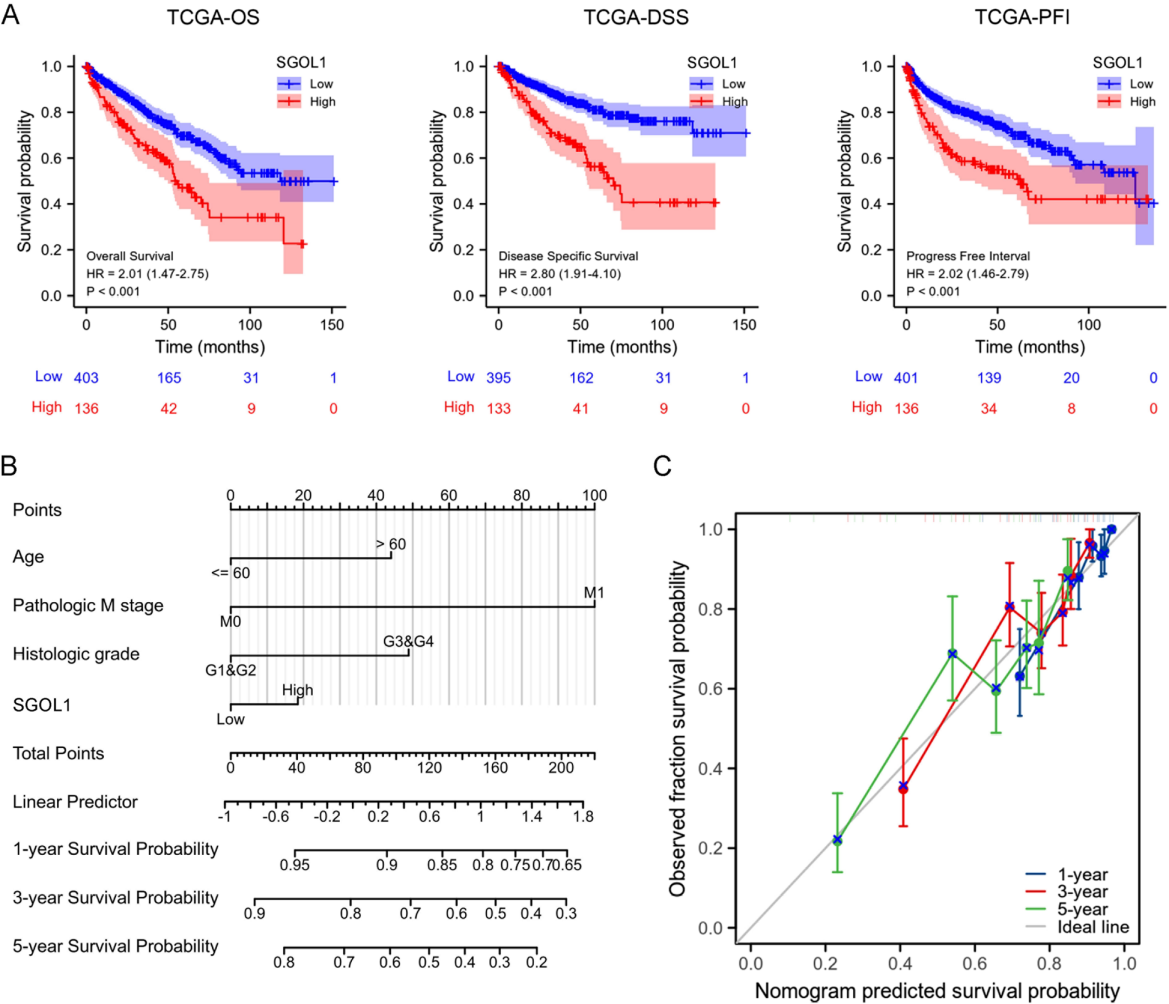
SGOL1 overexpression is linked to poor survival in ccRCC patients. **A** K–M survival analysis comparing OS, DSS, and PFI in patients with high and low SGOL1 expression. **B** A nomogram model was constructed for predicting the 1-, 3-, and 5-year OS of ccRCC patients with independent risk factors. **C** Calibration plots of the nomogram

Next, we explored the relationship between SGOL1 expression and the prognosis of ccRCC patients. Compared with those in the high-SGOL1-expression subgroup, we found that the low-SGOL1-expression subgroup in the TCGA-KIRC cohort had significantly favorable OS, PFI, and DSS (Fig. 3A). These results indicated that high SGOL1 expression levels were strongly associated with poor ccRCC patient outcomes. Afterward, we performed subgroup analyses based on clinical features. The results in Supplementary Fig. 3 show that the shorter OS of patients in the SGOL1 high-expression group was correlated with multiple factors, including age, gender, race (white), T stage (T3&T4), N stage (N0), M stage, pathologic stage (stage III&IV), and histologic grade (G3&G4). To further identify risk factors for ccRCC, univariate Cox regression analysis was performed. All variables significant in the univariate Cox regression analysis (*P* ≤ 0.05) were included in the multivariate Cox regression analysis. We found that age (HR = 1.687, CI = 1.098–2.592, *P* = 0.017), M stage (HR = 2.714, CI = 1.602–4.599, *P* < 0.001), histologic grade (HR = 1.700, CI = 1.033–2.799, *P* = 0.037), and SGOL1 expression (HR = 1.874, CI = 1.223–2.872, *P* = 0.004) were found to be independent risk factors for ccRCC (Supplementary Table 3). In addition, to better predict the prognosis of patients with ccRCC to guide clinical doctor treatment, a nomogram of OS at 1, 3, and 5 years based on the independent risk factor for multivariate Cox regression was constructed to assign the points of the variables (Fig. 3B). In addition, a calibration plot was generated to evaluate the prediction accuracy of the nomogram. The C-index of the model was 0.732 (0.713–0.752), which demonstrated that the nomogram had a moderately reliable ability to predict survival (Fig. 3C). These findings suggested that SGOL1 could serve as an independent prognostic risk factor associated with poor outcomes in ccRCC patients.

### Coexpression network and functional enrichment analysis of SGOL1

**Fig. 4:**
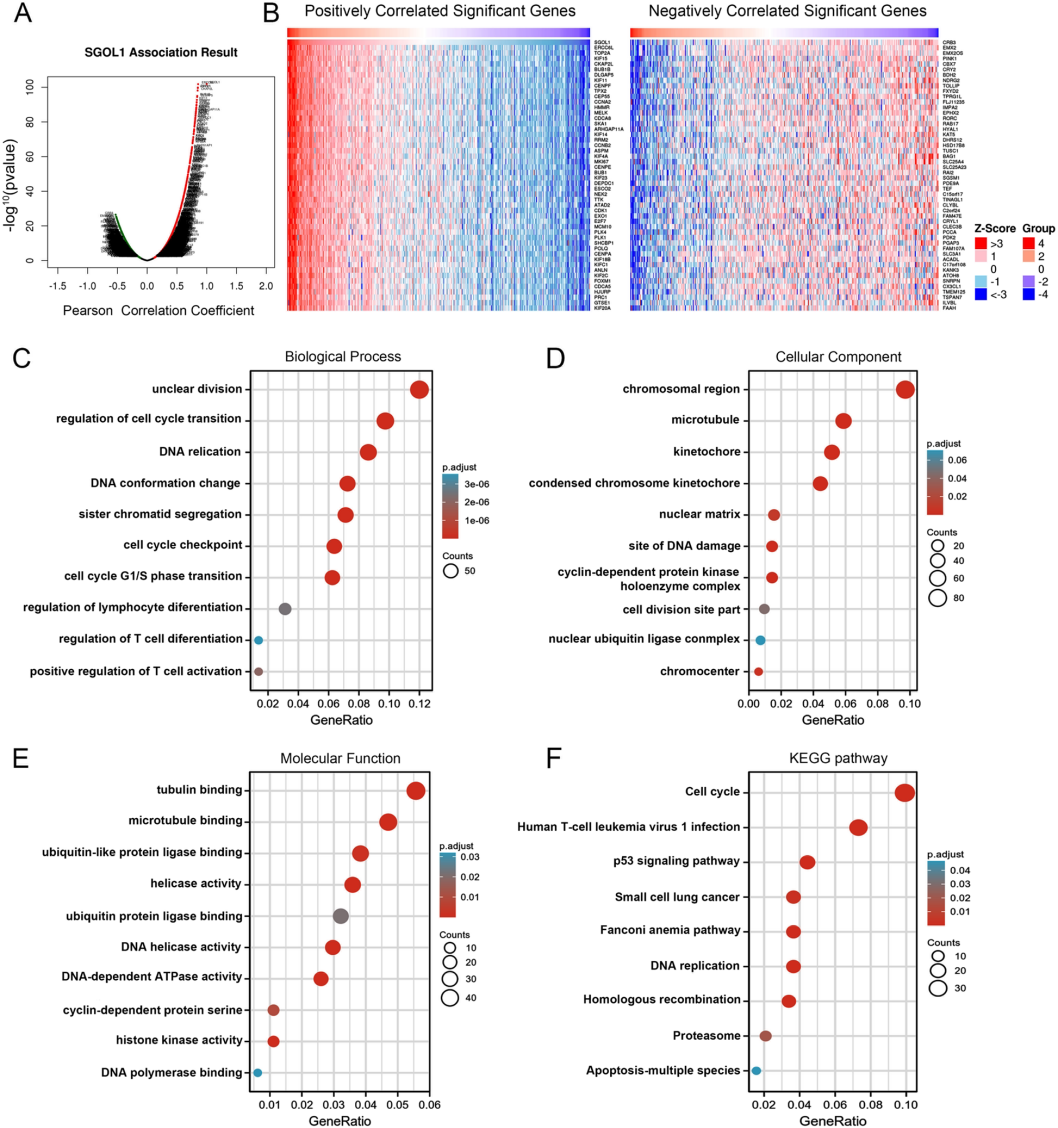
Coexpression networks and functional enrichment of SGOL1 in ccRCC. **A** The volcano map of the SGOL1 coexpressed genes is marked in red (positively correlated genes) and green (negatively correlated genes) according to the LinkedOmics database in the ccRCC cohort. **B** Heatmaps of the top 50 genes showing a significant positive or negative correlation with SGOL1. **C-F** The GO annotation and KEGG pathway enrichment data of SGOL1 based on the coexpression network are presented in a bubble plot

A coexpression network was constructed based on the TCGA-KIRC cohort to explore the biological importance and molecular mechanisms of SGOL1 in ccRCC. DEGs were identified and visualized by the LinkedOmics database and a volcano plot, respectively (Fig. 4A). A heatmap was generated to visualize the top 50 DEGs (Fig. 4B). These results suggested positive correlations between SGOL1 and ERCC6L, TOP2A, KIF15, KIF11, etc., and negative correlations between SGOL1 and EMX2, PINK1, CRB3, BDH2, etc. The top 1000 DEGs were subjected to GO and KEGG pathway enrichment analyses using the “clusterprofile” package in R software to identify the related signaling pathways and potential molecular functions. The main enriched biological processes (BPs) included nuclear division, regulation of cell cycle phase transition, sister chromatid segregation, and DNA replication (Fig. 4C). For the cellular component (CC) category, the main enriched GO terms were chromosomal region, microtubule, and kinetochore (Fig. 4D). The enriched molecular function (MF) terms included tubulin binding, microtubule binding, and helicase activity (Fig. 4E). Finally, KEGG pathway analysis revealed that SGOL1 and its correlated genes were significantly involved in the cell cycle, the P53 signaling pathway, homologous recombination, DNA replication, and apoptosis (Fig. 4F). These results suggest that SGOL1 may promote the malignant proliferation of ccRCC cells through cell cycle-related mitosis and DNA replication-related signaling pathways.

### Correlation between SGOL1 expression and immune infiltration in ccRCC

**Fig. 5:**
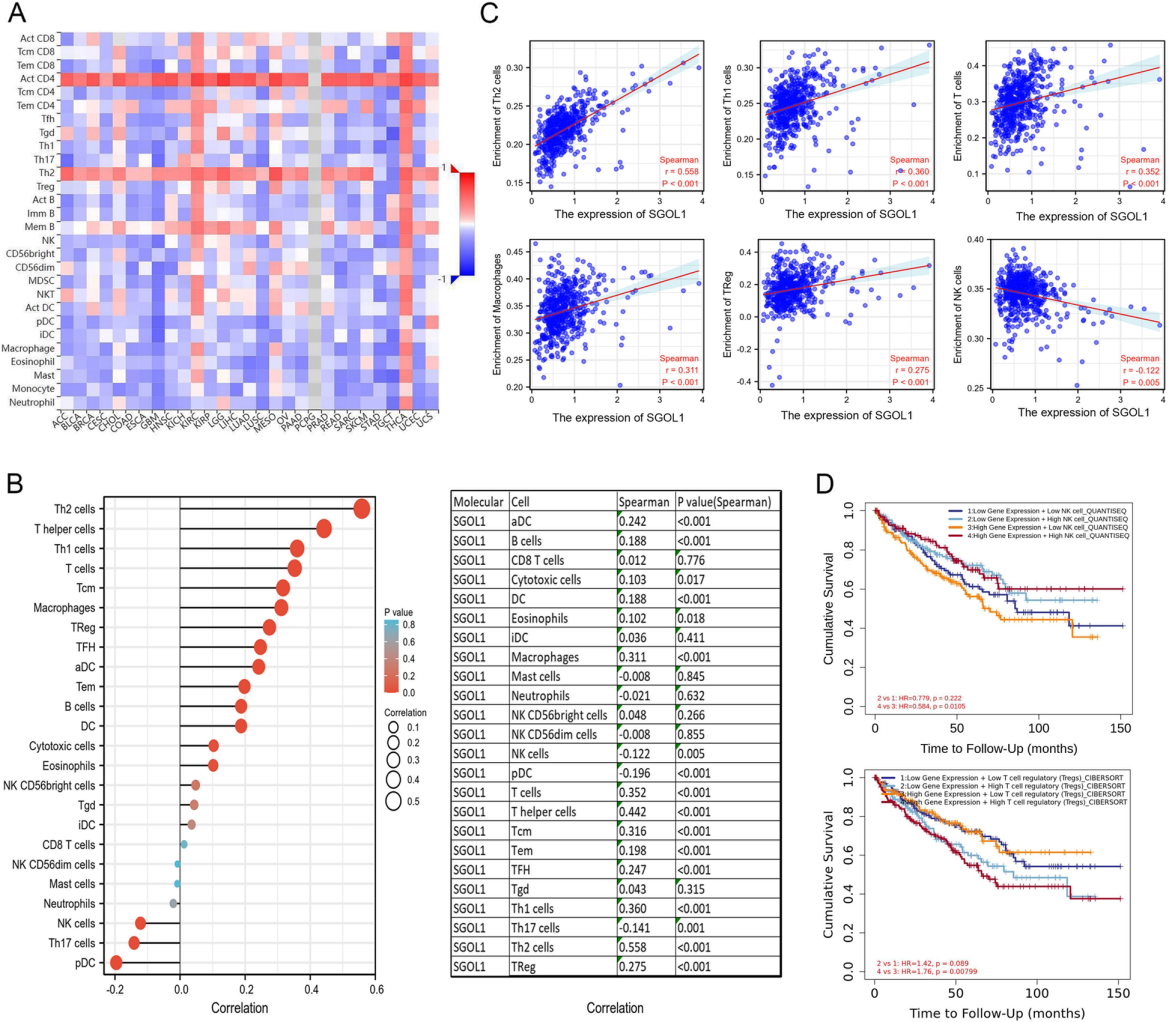
Correlation analysis of SGOL1 expression with immune cell infiltration in ccRCC (**A**) Heatmap of the associations between SGOL1 and 28 types of tumor-infiltrating lymphocytes (TILs) in various cancers. **B** Lollipop plot of the correlation analysis between SGOL1 expression and the enrichment of 24 types of infiltrating immune cells in ccRCC. SGOL1 was positively correlated with the level of Treg cell infiltration and negatively correlated with the level of NK cell infiltration. **C** Scatter plots of the correlation between SGOL1 expression and the infiltration of Th2 cells, Th1 cells, T cells, macrophages, Tregs, and NK cells. **D** Overall survival (OS) analysis of ccRCC patients stratified according to the abundance of infiltrating NK and Treg cells and high and low SGOL1 expression

Tumor tissue is composed of tumor cells and numerous immune cells infiltrating tumor cells; these immune cells can regulate tumor progression and affect patient prognosis and immunotherapy response [43, 44]. Clear cell renal cell carcinoma (ccRCC) has been proven to be a highly immune-infiltrating tumor in several clinical and genomic studies [45]. Hence, we first searched the TISIDB database to determine the link between SGOL1 and 28 tumor-infiltrating lymphocytes (TILs) in various cancers and found that SGOL1 was positively linked with TILs, particularly in ccRCC and thyroid carcinoma (THCA) (Fig. 5A). Spearman correlation analysis was performed to determine the correlation between the expression level of SGOL1 isolated from the TCGA-KIRC dataset and infiltration matrix data on 24 types of immune cells extracted from previously published research [46], and the results are presented in a lollipop plot after ssGSEA was performed with the R package GSVA and visualized with the R package ggplot2. We found that, compared with patients in the low-SGOL1-expression group, patients in the high-SGOL1-expression group presented significantly greater proportions of Th2 cells, T helper cells, Th1 cells, T cells, Tcm cells, macrophages, and Tregs (*P* < 0.05) and lower proportions of pDCs, Th17 cells, and NK cells (*P* < 0.05) (Fig. 5B); these changes in the proportions of infiltrating Th1 cells, Th2 cells, T cells, macrophages, Tregs and NK cells had great impacts on the tumor microenvironment and clinical prognosis of ccRCC patients [47, 48]. The scatter plots further revealed strong positive correlations between SGOL1 and Th2 cells (r = 0.558, *P* < 0.001), between SGOL1 and Th1 cells (r = 0.360, *P* < 0.001), between SGOL1 and T cells (r = 0.352, *P* < 0.001), between SGOL1 and macrophages (r = 0.311, *P* < 0.001), and between SGOL1 and regulatory T cells (Tregs) (r = 0.275, *P* < 0.001) and between SGOL1 and natural killer (NK) cells (r =—0.122, *P* = 0.005) (Fig. 5C). Natural killer (NK) cells are innate lymphoid cells with powerful anti-inflammatory and antitumor activities. NK cells express a range of germline-encoded receptors that eliminate transformed cells and retain normal healthy cells, thus playing key roles in inhibiting tumorigenesis, controlling tumor growth, and mediating powerful antimetastatic effects. However, due to the presence of immunosuppressive microenvironments that affect NK cell function, some tumors can develop tolerance to NK cell-mediated killing [49–51]. According to previous studies, regulatory T cells (Tregs) have a high degree of immunosuppressive function and play an important role in maintaining self-tolerance and immune homeostasis; however, in malignant tumors, Tregs, an immunosuppressive subset of CD4 + T cells, can exhibit protumor activity by inhibiting the activation and proliferation of effector T cells, thus limiting the autoimmune response [52, 53]. Therefore, the increased infiltration of Treg cells and decreased infiltration of NK cells are closely related to the immunosuppressive tumor microenvironment (TME) and poor prognosis of patients with ccRCC [54–56]. Considering the results above, we investigated whether the poor prognosis of ccRCC patients mediated by Treg cells and NK cells was affected by SGOL1 expression. As shown in Fig. 5D, we found meaningful correlations between elevated Treg cell counts and decreased NK cell counts and unfavorable clinical outcomes in the high-SGOL1-expression group; however, there was no significant difference in the low-SGOL1-expression group. Collectively, these results suggest that high SGOL1 expression may act as an immunoregulatory factor contributing to ccRCC malignant progression and poor prognosis by creating an immunosuppressive TME.

### SGOL1 could predict immune checkpoint inhibitor efficacy

**Fig. 6:**
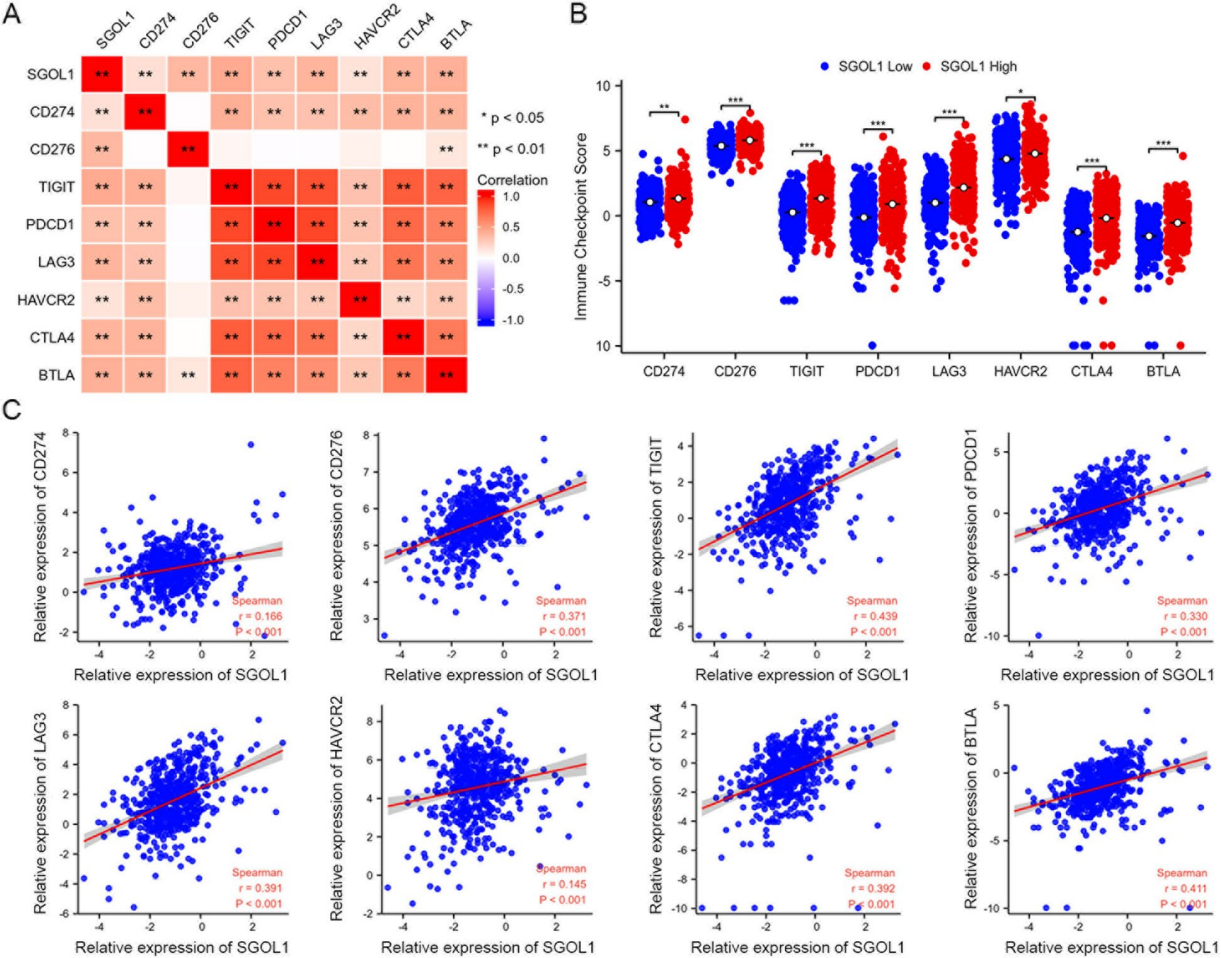
Correlation between SGOL1 expression and immune checkpoint inhibitor (ICI) expression in ccRCC patients. **A** Correlation heatmap of SGOL1 and immune checkpoint inhibitor (ICI) expression in the TCGA-KIRC cohort. Red indicates a positive correlation; blue indicates a negative correlation; the Spearman correlation coefficient is represented by the color intensity. **B** Compared with that in the low-SGOL1-expression group, the expression of immune checkpoint inhibitors (ICIs) (CD274, CD276, TIGIT, PDCD1, LAG3, HAVCR2, CTLA4, and BTLA) was significantly upregulated in patients with high SGOL1 expression, *n* = 265. **C** Scatter plots illustrate that SGOL1 expression was positively related to ICI expression. (*P < 0.05, **P < 0.01, ***P < 0.001)

The effectiveness of immunotherapy depends on adequate immune cell infiltration in the TME and sufficient expression of immune checkpoint inhibitors (ICIs) [57]. Therefore, the correlations between the expression of SGOL1 and immune checkpoint inhibitors (ICIs), including CD274 (PD-L1), CD276 (B7-H3), TIGIT, PDCD1, LAG3, HAVCR2, CTLA4, and BTLA, were evaluated (Fig. 6A). ICI expression was compared between the high-SGOL1-expression and low-SGOL1-expression groups, and the results suggested that ICIs were significantly highly expressed in the high-SGOL1-expression group, indicating that SGOL1 might be correlated with the efficacy of immune checkpoint inhibitors (ICIs) because of the consistency of SGOL1 and ICI expression (Fig. 6B). Moreover, scatter plots were constructed to further illustrate the correlation between SGOL1 and commonly studied ICIs, CD274 (PD-L1), CD276 (B7-H3), TIGIT, PDCD1, LAG3, HAVCR2, CTLA4, and BTLA, in ccRCC patients, and the results demonstrated a strong positive correlation between SGOL1 and ICI expression (Fig. 6C). Taken together, these results suggest that SGOL1 might serve as an indicator of ICI efficacy in ccRCC patients and a predictor of the clinical application of ICI therapy to better assist clinicians in treatment; however, additional experimental validation is needed.

### SGOL1 was found to be targeted and regulated by miR-23b-3p in ccRCC

**Fig. 7:**
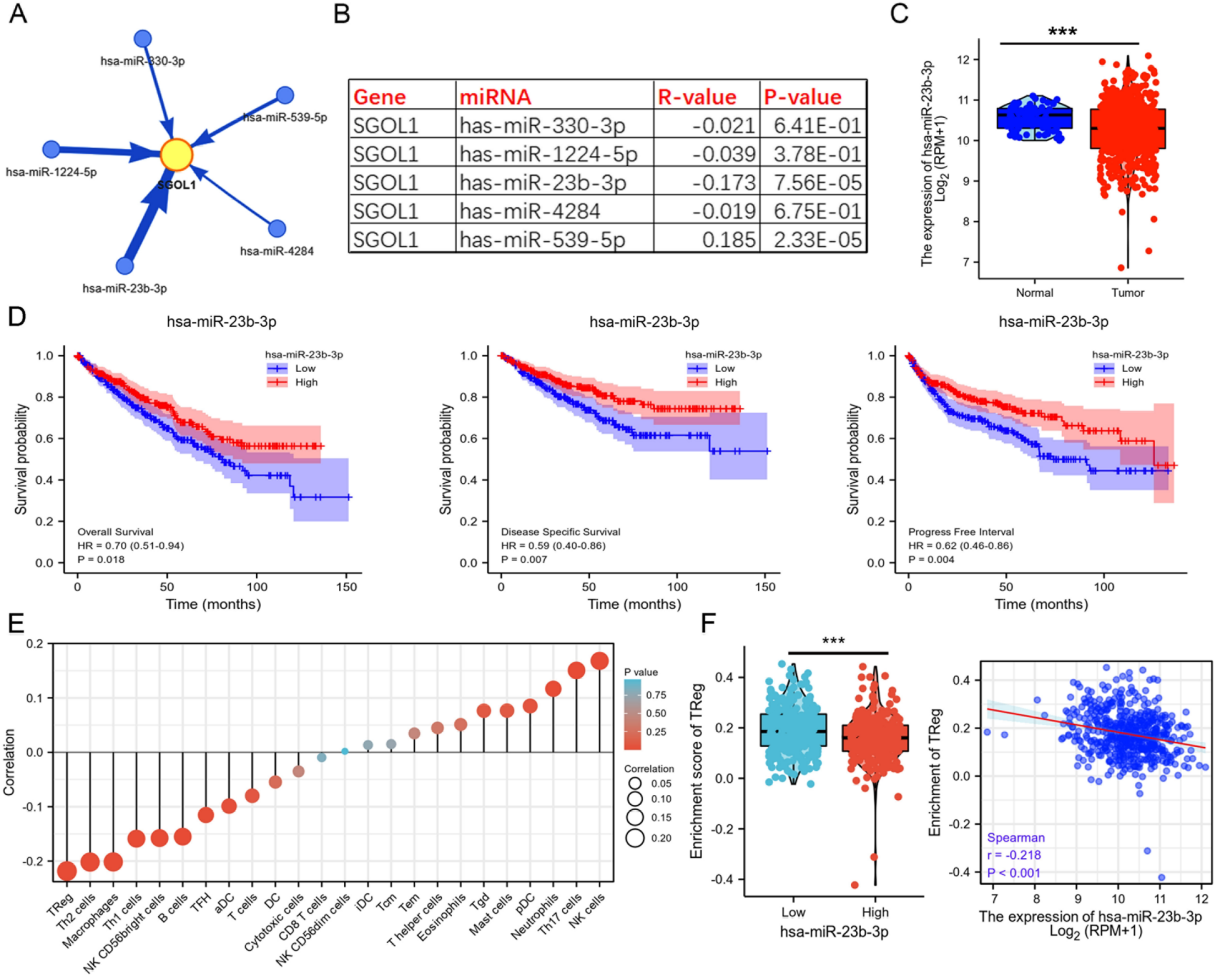
SGOL1 was found to be potentially targeted by miR-23b-3p in ccRCC. **A** Five potential binding miRNAs targeting SGOL1 based on the GSCA database. **B** Correlation analysis between the five candidate miRNAs and SGOL1 expression in ccRCC. The R-value represents the Spearman correlation coefficient. **C** Comparison of miR-23b-3p expression between ccRCC tissues and peritumoral normal tissues (Normal = 71, Tumor = 545). **D** Survival analysis (OS, DSS, and PFI) confirmed that miR-23b-3p upregulation was correlated with favorable survival in ccRCC patients. (E) Bubble plot of the correlation between the expression of miR-23b-3p and 24 types of immune cells in ccRCC. (F) Correlation analysis of miR-23b-3p expression and Treg cell infiltration by box and scatter plots (Low = 270, High = 271). (***P < 0.001)

To thoroughly examine the upstream regulatory framework, we obtained the most likely potential miRNAs that bind to SGOL1 using the GSCA database. The results revealed miR-330-3p, miR-1224-5p, miR-23b-3p, miR-4284, and miR-539-5p as potential upstream regulators of SGOL1, in which only miR-23b-3p was meaningfully negatively correlated with SGOL1 (r = -0.173, *P* < 0.001) (Fig. 7A, B). In addition, miR-23b-3p was apparently expressed at a lower level in ccRCC tissues than in normal kidney tissues (Fig. 7C). Moreover, Kaplan–Meier analysis suggested that ccRCC patients with low expression of miR-23b-3p were associated with a worse prognosis (OS, DSS, and PFI) (Fig. 7D). Additionally, correlation analysis of immune cell infiltration showed that the expression of miR-23b-3p was strongly negatively correlated with the infiltration of Treg cells, Th1 cells, and Th2 cells in ccRCC but was significantly positively correlated with the infiltration of NK cells, which was consistent with the above results concerning the immune infiltration of SGOL1 (Fig. 7E). Specifically, we found that the enrichment of Treg cell infiltration in the high-miR-23b-3p group was dramatically decreased. Moreover, scatter plots were constructed to illustrate further the negative correlation between miR-23b-3p expression and Treg infiltration (r = -0.218, *P* < 0.001) (Fig. 7F). Combined with the previous finding that SGOL1 was highly expressed and positively correlated with Treg enrichment in ccRCC, we believe that miR-23b-3p is a vital upstream molecule that targets SGOL1 and regulates SGOL1 expression, thus affecting Treg infiltration enrichment in ccRCC tissues. Overall, we provide evidence that miR-23b-3p acts as a key regulator that influences SGOL1 expression and function in ccRCC.

### Identification of a potential SNHG17/PVT1/ZMIZ1-AS1-miR-23b-3p-SGOL1 axis in ccRCC

**Fig. 8:**
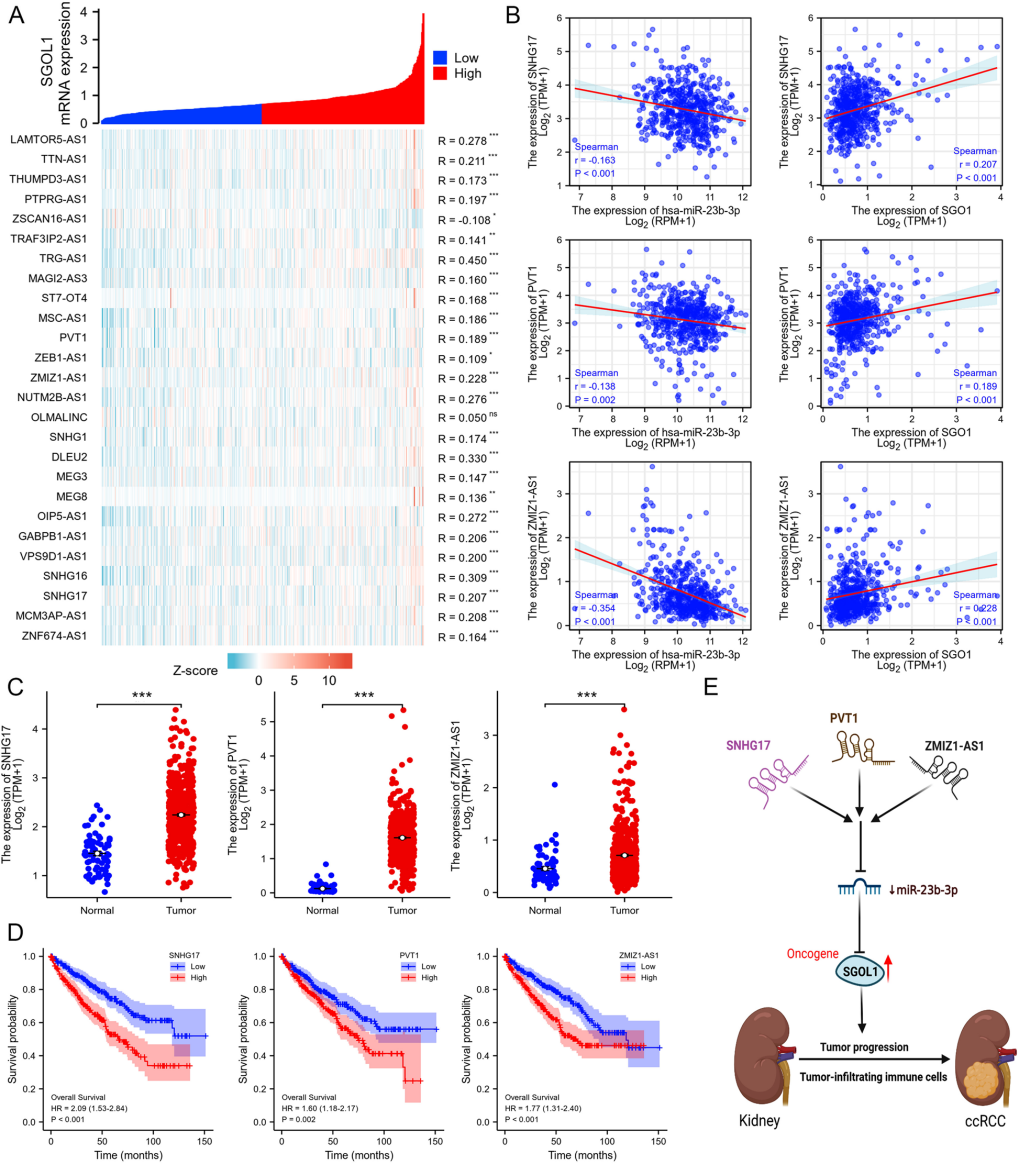
SNHG17, PVT1, and ZMIZ1-AS1 were identified as potential upstream lncRNAs of miR-23b-3p in ccRCC. **A** Coexpression heatmap of potential candidate lncRNAs positively correlated with SGOL1. **B** Scatter plot of the correlations between lncRNAs (SNHG17, PVT1, and ZMIZ1-AS1) and miR-23b-3p and SGOL1. **C** The expression patterns of SNHG17, PVT1, and ZMIZ1-AS1 in ccRCC tissues (*n* = 541) and peritumoral normal tissues (*n* = 72). **D** OS analysis revealed that SNHG17, PVT1, and ZMIZ1-AS1 upregulation was linked to a worse prognosis in ccRCC patients. **E** Schematic representations of the SNHG17/PVT1/ZMIZ1-AS1/miR-23b-3p/SGOL1 axis in the carcinogenesis of ccRCC. (*P < 0.05, **P < 0.01, ***P < 0.001)

LncRNAs usually serve as competing endogenous RNAs (ceRNAs), binding to miRNAs to prevent them from repressing their target mRNAs [58]. Therefore, the potential candidate lncRNAs should be positively correlated with SGOL1 and negatively correlated with miR-23b-3p. We subsequently investigated the potential lncRNAs that target miR-23b-3p using the starBase website and were positively linked with SGOL1 expression using correlation analysis based on TCGA-KIRC data; we found that only 26 lncRNAs were involved (Fig. 8A). Considering the ceRNA hypothesis, lncRNA expression should negatively correlate with miR-23b-3p expression in ccRCC. According to the correlation analysis of the 26 candidate lncRNAs with miR-23b-3p, only SNHG17, PVT1, and ZMIZ1-AS1 were found to be positively correlated with SGOL1 and negatively correlated with miR-23b-3p in the TCGA-KIRC cohort (Fig. 8B). Accordingly, the expression of the lncRNAs SNHG17, PVT1, and ZMIZ1-AS1 was upregulated in ccRCC tissues compared with that in peritumoral normal tissues, and high expression of these lncRNAs (SNHG17, PVT1, and ZMIZ1-AS1) was associated with poor ccRCC patient prognosis (Fig. 8C, D). Moreover, the associations between lncRNAs (SNHG17, PVT1, and ZMIZ1-AS1) and immune cell infiltration were also investigated, and the results suggested that there was a strong positive correlation between Treg cell infiltration and lncRNA (SNHG17, PVT1, and ZMIZ1-AS1) expression. Tregs, as tumor immunosuppressive cells, often contribute to poor clinical outcomes in patients with ccRCC (Supplementary Fig. 4). Overall, we identified a potential SNHG17/PVT1/ZMIZ1-AS1-miR-23b-3p-SGOL1 axis, which is related to the diagnosis, clinical prognosis, and malignant progression of ccRCC (Fig. 8E).

### The potential effect of SGOL1 on ccRCC cell proliferation

**Fig. 9:**
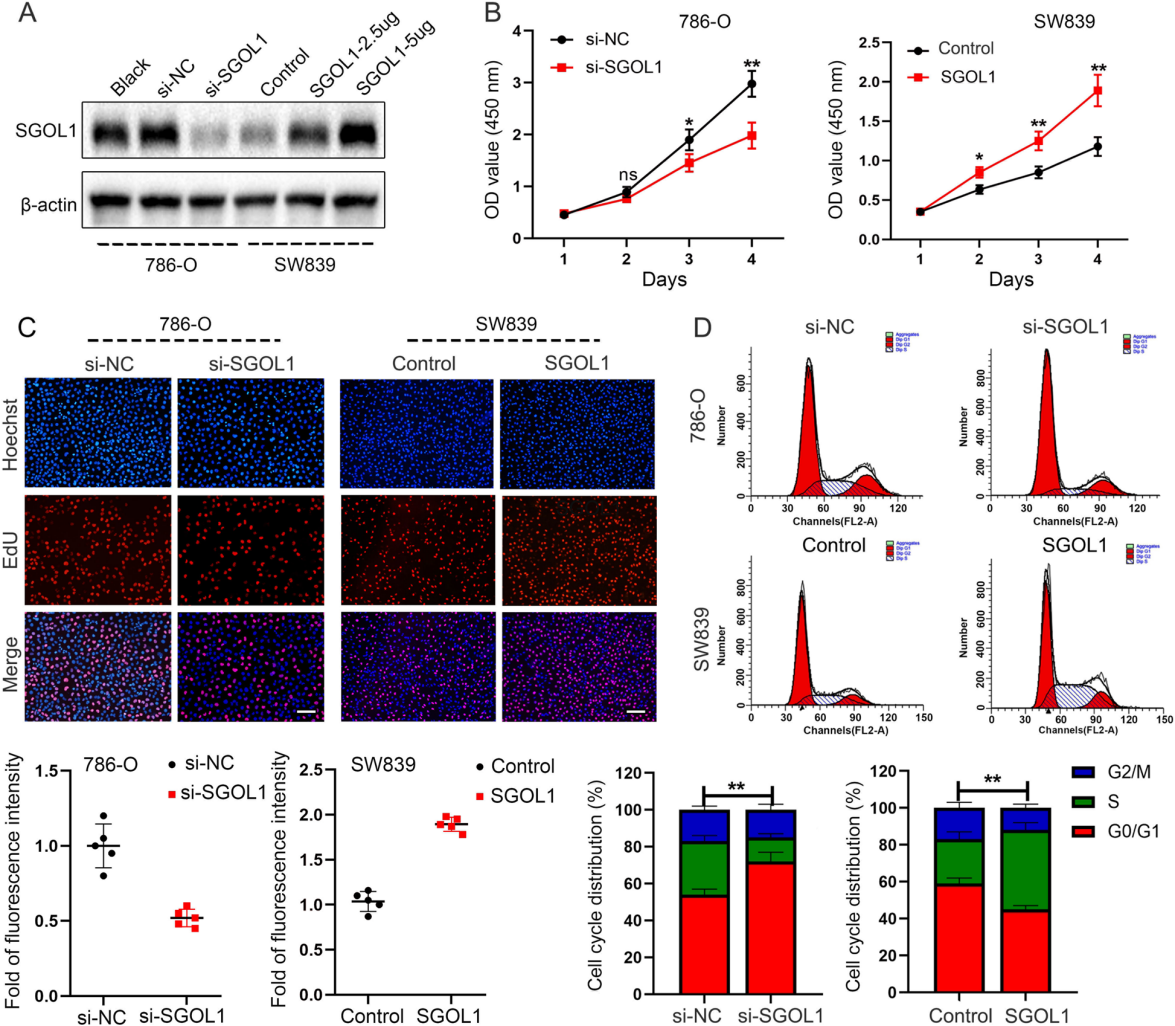
SGOL1 promoted cell proliferation in ccRCC. **A** The efficiency of SGOL1 interference and overexpression in ccRCC cells was validated by western blot analysis. **B** A CCK-8 assay was conducted to assess ccRCC cell viability. (C) An EdU staining assay was performed to assess the DNA replication capacity of ccRCC cells. *n* = 5, Scale bar: 100 μm. (D) Flow cytometry analysis of the cell cycle distribution of ccRCC cells. (**P < 0.01, ***P < 0.001)

The above experiments revealed that SGOL1 was upregulated in ccRCC tissues and cell lines, with 786-O cells exhibiting the highest expression and SW839 cells showing the lowest. Hence, we knocked down SGOL1 by transfecting 786-O cells with SGOL1 siRNA and overexpressed SGOL1 by transfecting SW839 cells with an SGOL1 overexpression plasmid to investigate the potential biological function of SGOL1 in ccRCC cells (Fig. 9A). Combined with the gene functional enrichment analysis results, these findings revealed that SGOL1 plays a vital role in regulating the malignant proliferation of ccRCC cells. The results of the CCK-8 and EdU staining assays indicated that the upregulation of SGOL1 promoted cell proliferation, whereas SGOL1 inhibition suppressed cell proliferation by altering cell viability and DNA replication capacity (Fig. 9B, C). As shown in Fig. 4C, SGOL1 may act as a cell cycle checkpoint and regulate the G1/S transition. Flow cytometry assays revealed that the upregulation of SGOL1 significantly decreased the number of cells in the G0/G1 phase and increased the number of cells in the S phase, whereas the downregulation of SGOL1 arrested the cells in the G0/G1 phase, which suggested that SGOL1 could promote cell proliferation by accelerating cell cycle progression (Fig. 9D). Collectively, these data indicated that SGOL1 upregulation could increase the proliferative capacity of ccRCC cells.

### Effect of SGOL1 on cell migratory capacity and invasiveness in ccRCC

**Fig. 10:**
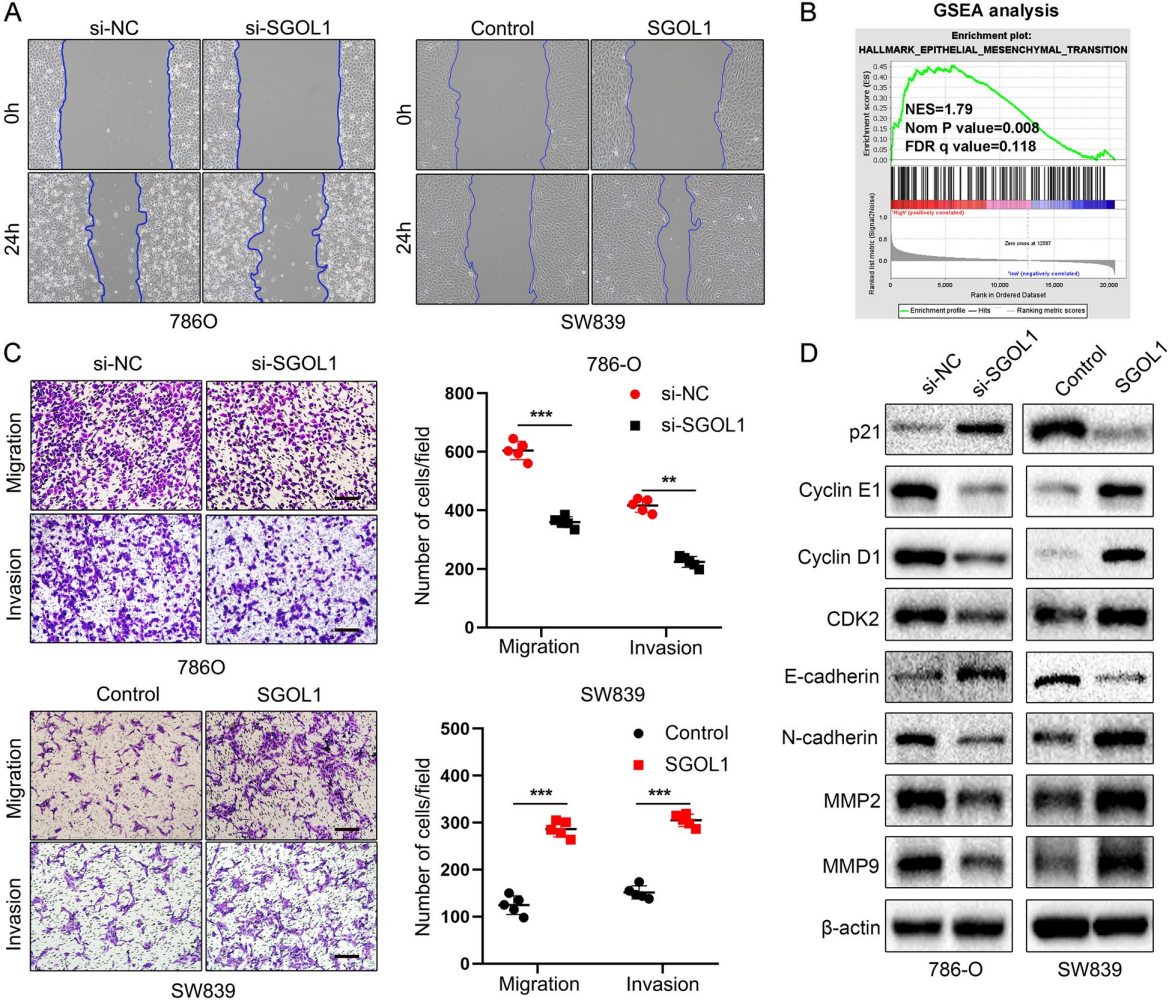
SGOL1 promoted ccRCC cell migration and invasion. **A** A wound healing assay was conducted to assess the migration of 786-O and SW839 cells. **B** GSEA results of “hallmark epithelial–mesenchymal transition” enrichment in the high-SGOL1-expression group. **C** Representative images of Transwell migration and invasion in SGOL1-KD 786-O cells and SGOL1-OE SW839 cells. *n* = 5, Scale bar = 100 μm. The results of the quantitative analysis are shown as mean ± SD on the right. **D** The protein expression of p21, Cyclin D1, Cyclin E1, CDK2, E-cadherin, N-cadherin, MMP2, and MMP9 in SGOL1-KD 786-O cells and SGOL1-OE SW839 cells was assessed via western blotting. (**P < 0.01, ***P < 0.001)

GSEA was performed to assess the potential biological effect of SGOL1 on ccRCC and the signaling pathways through which SGOL1 exerts its effects, and the results suggested that the “hallmark epithelial–mesenchymal transition” (EMT) was enriched in patients with relatively high SGOL1 expression (Fig. 10B). The EMT is an important process associated with tumor metastasis. Wound healing and Transwell assays with or without Matrigel were also conducted to assess the potential function of SGOL1 in the EMT process. A wound healing assay revealed that SGOL1 downregulation decreased the pace of wound closure in 786-O cells, while SGOL1 upregulation increased the wound closure rate in SW839 cells (Fig. 10A). Transwell assays, including migration and invasion assays, showed that upregulation of SGOL1 in SW839 cells promoted, while downregulation of SGOL1 in 786-O cells inhibited, the migratory capacity and invasion of ccRCC cells (Fig. 10C). We also detected the expression of EMT- and proliferation-related biomarkers via western blotting. As expected, we found that the expression of proliferation-related proteins such as Cyclin E1, Cyclin D1, and CDK2 was upregulated after SGOL1 overexpression, while the expression of p21, a classical proliferative suppressor gene, was downregulated. Moreover, SGOL1 overexpression accelerated the EMT process by downregulating the epithelial marker E-cadherin and upregulating the mesenchymal marker N-cadherin and the matrix metallopeptidases MMP2 and MMP9 in SW839 cells. The opposite results were observed in 786-O cells after SGOL1 inhibition. (Fig. 10D). Collectively, these data demonstrated that SGOL1 acts as an oncogene in ccRCC by promoting cell proliferation, migratory capacity and invasion.

## Discussion

The most common subtype of kidney cancer among the several known forms is ccRCC, which accounts for more than 80% of all kidney cancer cases [2]. The 5-year survival rate of patients with early-stage ccRCC is as high as 90%, while that of patients with advanced-stage and metastatic disease drops to 60% and 10%, respectively [59]. Moreover, given the limited effects of chemotherapy and the relative insensitivity of ccRCC to radiotherapy, complete tumor extirpation is still the first-line therapy for the clinical treatment of ccRCC. Despite substantial advancements in molecular targeted therapy for the treatment of advanced ccRCC, the outcomes are still poor, and only a few people benefit from this therapy [60]. In recent years, immunotherapy involving immune checkpoint (PD-1, CTLA4) inhibitors has emerged as an effective treatment option for advanced ccRCC [61]. Moreover, studies published in Lancet Oncology also confirmed the superiority of immunotherapy over antivascular drugs in the treatment of metastatic kidney cancer. The success of cancer immunotherapy via immune checkpoint inhibitors has provided new insights into cancer treatment for ccRCC. Unfortunately, more than half of treated patients do not achieve clinical benefit [62, 63]. Therefore, identifying reliable biomarkers that predict clinical prognosis and immunotherapy efficacy in patients with ccRCC who may benefit more from immunotherapy or targeted therapy is urgently needed.

SGOL1, a conserved protein and a Shugoshin family member, was first identified in 2005 as a regulator of chromosomal segregation [64]. Previous studies have confirmed that SGOL1 preserves chromosomal integrity by preventing early sister chromatid separation and maintaining microtubule tension. Therefore, SGOL1 plays an important role in maintaining cell proliferation [65, 66]. Using the HPA website, we found that SGOL1 was located mainly in the cytoplasm of cancer cells, which is consistent with its functional role in cells (Supplementary Fig. 1B). Moreover, certain reports have suggested that SGOL1 functions as an oncogene that promotes tumor progression. For example, SGOL1 promotes prostate cancer progression by promoting cell proliferation and accelerating the EMT process [21, 67]. Additionally, SGOL1 is regarded as a diagnostic and prognostic biomarker for various cancers, such as colorectal cancer and hepatocellular carcinoma [68, 69]. Nevertheless, few studies have focused on the potential biological functions and regulatory network of SGOL1 in ccRCC, and the underlying molecular mechanism through which SGOL1 regulates ccRCC progression is poorly understood. Hence, a series of functional experiments combined with bioinformatics analyses were performed to clarify the specific effects of SGOL1 on ccRCC, as well as its prognostic and diagnostic value and oncogenic role in ccRCC from multiple perspectives. This article comprehensively elucidated the specific role of SGOL1 in the carcinogenesis and progression of ccRCC, which provides a new approach for the future clinical diagnosis and treatment of ccRCC.

In this study, through the analysis of SGOL1 expression levels in various types of cancer, we observed that SGOL1 expression was significantly upregulated in almost all tumor tissues, including ccRCC tissues, compared with normal tissues, although to different degrees. Western blot analysis combined with immunohistochemical staining of ccRCC tissue chips suggested that SGOL1 was upregulated in ccRCC tissues and cells. Moreover, increased SGOL1 expression was strongly associated with adverse clinical pathological characteristics, including advanced cancer stage, increased lymphatic metastasis, and increased tumor grade, and predicted worse clinical survival (OS, DSS, PFI) in ccRCC patients. Based on univariate and multivariate Cox regression analyses and a nomogram model with suitable calibration, we successfully constructed an SGOL1-based prognostic prediction system, which suggested that SGOL1 could serve as an oncogene and a reliable and independent prognostic biomarker of ccRCC. However, further studies are needed to validate these findings further.

To investigate the potential biological functions of SGOL1 in ccRCC, a coexpression network of SGOL1 was constructed, and the top 1000 DEGs in the high-SGOL1-expression and low-SGOL1-expression groups were screened out and subjected to functional enrichment analysis. The results indicated that SGOL1 was located mainly in the chromosomal region and microtubules and was positively correlated with cell proliferation by regulating nuclear division, cell cycle transition, DNA replication, and the p53 signaling pathway. Mutations in the p53 signaling pathway can cause uncontrolled cell proliferation and tumorigenesis and are regarded as hallmarks of cancer [70, 71]. Previous research has confirmed that SGOL1 inhibition suppresses the progression of various tumors, such as neuroblastoma and hematological malignancies, by inducing cell cycle arrest and proliferation inhibition [19, 72]. In this study, experimental validation via gain- and loss-of-function assays demonstrated that SGOL1 overexpression promoted cell proliferation by increasing cell viability, colony formation ability, cell cycle progression, and DNA replication capacity, while SGOL1 inhibition had the opposite effects. In addition, GSEA strongly suggested that SGOL1 promoted ccRCC metastasis by accelerating the epithelial–mesenchymal transition (EMT), which contributes to this process and is an essential step in cancer metastasis [73]. Functional experiments also confirmed that SGOL1 inhibition suppressed the migratory and invasive capacity of ccRCC cells, while SGOL1 overexpression enhanced this capacity. Overall, the results indicated that SGOL1 had a positive effect on ccRCC progression by promoting cell proliferation, invasion, and metastasis in vitro.

Tumor development, growth, and metastasis are influenced by both the malignant properties of cancer cells and the immunosuppressive TME [74]. ccRCC is characterized by both high immune infiltration and immunosuppression, as indicated by elevated immune cell infiltration and upregulated expression of immune checkpoint surface biomarkers [45]. Hence, the correlations between SGOL1 expression and immune cell infiltration and between SGOL1 expression and immune checkpoint expression were investigated in this research. By conducting a pan-cancer analysis, we found that SGOL1 was positively associated with immune cell infiltration in ccRCC. We further analyzed 24 immune-associated gene sets representing diverse immune cell types in ccRCC and their correlation with SGOL1 expression and quantified the activity or enrichment levels of immune cells using the ssGSEA algorithm. SGOL1 was positively correlated with the infiltration of Th2 cells, Th1 cells, T cells, macrophages, and Tregs but negatively correlated with the infiltration of NK cells. NK cells act as tumor immune surveillance cells to inhibit tumor progression, while Treg cells create an immunosuppressive microenvironment to promote tumor progression [75, 76]. Regulatory T (Treg) cells are one of the major immunosuppressive cell types in cancer and potential targets for immunotherapy. Moreover, Treg cells function as manipulators, creating an immunosuppressive TME in ccRCC through multiple pathways. For example, Treg cells highly enriched in the ccRCC TME but not in normal kidney tissues could suppress the activation and function of effector T cells, create an immunosuppressive microenvironment, and promote tumor immune escape [77, 78]. Moreover, an increase in Treg infiltration was linked to adverse clinicopathological characteristics and worse survival in ccRCC patients [79]. In this study, we also demonstrated that lower NK cell infiltration and higher Treg cell infiltration correlated with a worse prognosis in ccRCC patients with high SGOL1 expression. The efficacy of immunotherapy not only requires adequate immune cell infiltration in the ccRCC TME but also depends on the sufficient expression of immune checkpoint inhibitors [80]. CcRCC was historically one of the first and most responsive malignancies to immune checkpoint inhibitors (ICIs). ICIs significantly improved the prognosis of patients with advanced ccRCC [81]. The expression of immune checkpoint inhibitors, such as CD274 (PD-L1), CD276, TIGIT, PDCD1 (PD-1), LAG3, HAVCR2, CTLA4, and BTLA, was compared between the high-SGOL1-expression and low-SGOL1-expression groups of ccRCC patients, and the results suggested that immune checkpoint inhibitor expression was significant and highly expressed in the high-SGOL1-expression group. High levels of cosuppressor receptors, including CTLA-4, PD-L1, and TIGIT (T-cell immunoreceptor with Ig and ITIM domains), on the surface of Treg cells exert immunosuppressive effects by interacting with ligands on their target cells [55, 82]. Taken together, these findings indicate that SGOL1 promotes the malignant progression of ccRCC by constructing an immunosuppressive microenvironment, increasing the infiltration of Treg cells, and upregulating the expression of immune checkpoint inhibitors. Moreover, SGOL1 can serve as an indicator of the therapeutic efficacy of ICIs. However, additional experimental analyses are needed to validate the importance of SGOL1 in regulating Treg infiltration and ICI efficacy.

In recent years, many lncRNAs have been found to play crucial roles in tumor progression and can regulate the malignant behavior of tumors by affecting various biological processes, such as cell proliferation, metastasis, chemo-/radioresistance, and immune responses [83–85]. LncRNAs function as ceRNAs and are defined as miRNA sponges that compete with targeted miRNAs indirectly, thus downregulating miRNAs and altering the expression of downstream target genes [86]. Numerous studies have confirmed that the lncRNA–miRNA-mRNA network has been widely characterized in a broad spectrum of biological processes in ccRCC and may be a biomarker for the early diagnosis of ccRCC and a potential therapeutic target [58]. Here, we constructed a ceRNA regulatory network of SGOL1 in ccRCC via bioinformatics analysis. First, expression correlation and survival analyses based on the GSCA and TCGA databases revealed miR-23b-3p as the highest potential upstream miRNA of SGOL1 in ccRCC, which was significantly negatively correlated with SGOL1. Specifically, miR-23b-3p was downregulated in ccRCC tissues compared with normal tissues, and miR-23b-3p upregulation was linked to favorable prognosis in ccRCC patients. Moreover, there was a strong negative correlation between miR-23b-3p expression and Treg infiltration and a positive correlation between miR-23b-3p and NK cell infiltration, consistent with the above results. Therefore, we propose that miR-23b plays an inhibitory role in ccRCC progression by suppressing the oncogenic role of SGOL1. Next, we predicted the upstream lncRNAs of the miR-23b-3p/SGOL1 axis in ccRCC. Based on the starBase database and expression correlation analysis of SGOL1, three lncRNAs, SNHG17, PVT1, and ZMIZ1-AS1, were screened for further validation. The results suggested that SNHG17, PVT1, and ZMIZ1-AS1 were positively correlated with SGOL1 but negatively correlated with miR-23b-3p in ccRCC. In addition, SNHG17, PVT1, and ZMIZ1-AS1 were upregulated in ccRCC tissues compared with normal tissues and strongly positively associated with Treg infiltration. ccRCC patients with relatively high SNHG17, PVT1, and ZMIZ1-AS1 expression were found to have a poor prognosis. Finally, we identified a potential SNHG17/PVT1/ZMIZ1-AS1-miR-23b-3p-SGOL1 axis that regulates the progression of ccRCC. SGOL1 may serve as a prognostic biomarker and a promising therapeutic target for ccRCC.

In summary, our research demonstrated that SGOL1 is upregulated in ccRCC and is positively correlated with adverse clinicopathological characteristics and unfavorable prognosis. SGOL1 could serve as an independent prognostic predictor and a reliable diagnostic marker for ccRCC. In addition, SGOL1 expression was significantly positively correlated with Treg infiltration and immune checkpoint inhibitor expression. In addition, the ceRNA network of the SNHG17/PVT1/ZMIZ1-AS1-miR-23b-3p-SGOL1 axis might offer novel perspectives for the immunotherapy of ccRCC patients.

### Supplementary Information

**Supplementary material 1.**

**Supplementary material 2.**

**Supplementary material 3.**

**Supplementary material 4.**

## Data Availability

Publicly available datasets were analyzed in this study. The datasets supporting the conclusions of this article are available in the TCGA (https://portal.gdc.cancer.gov/) and GEO (https://www.ncbi.nlm.nih.gov/geo/) databases. Further inquiries can be directed to the corresponding author.
