# Supplementary material for: Prognosis and biological function of SGOL1 in clear cell renal cell carcinoma: a multiomics analysis
Source: BMC Med Genomics. 2024 Feb 21;17:60. doi: 10.1186/s12920-024-01825-7 (PMC10882763; doi:10.1186/s12920-024-01825-7)
Supplement: Supplementary file 1 — Supplementary material 1. [file 12920_2024_1825_MOESM1_ESM.pdf]

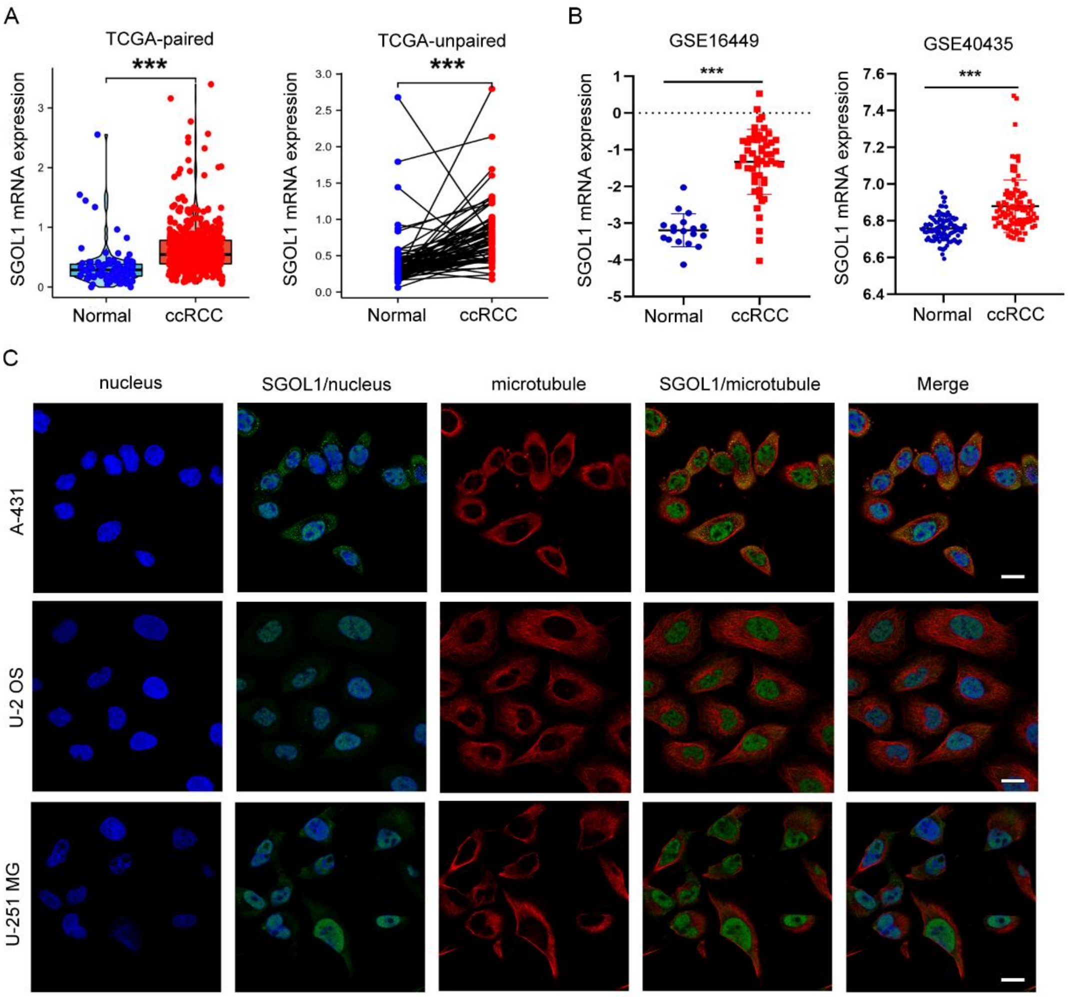

Supplementary Fig 1. The expression pattern and cellular localization of SGOL1 in ccRCC tissues and tumor cells (A) The paired and unpaired analysis of SGOL1 mRNA expression in TCGA-KIRC. (B) SGOL1 expression patterns in tumor and normal tissues were analyzed in GEO datasets (GSE16449 and GSE40435). (C) Representative immunofluorescence images of SGOL1 protein (Green) subcellular localization with DAPI (Blue) for nuclear staining and microtubule (Red) for the cytoskeleton in A431, U-2 OS, and U-251 MG cells from the HPA website. A431 is a human skin carcinoma cell line; U-2 OS is an osteosarcoma cell line; U-251 MG is a human glioblastoma cell. Scale bar: 20 µm. (***P < 0.001)

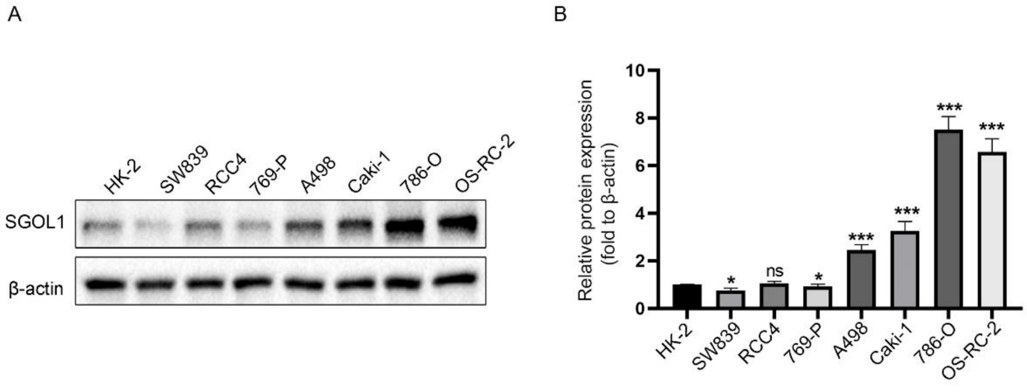

Supplementary Fig 2. SGOL1 expression in ccRCC cell lines and HK-2 cells. (A) The relative expression of SGOL1 protein was detected by western blotting in different ccRCC cell lines and HK-2 cells. β-actin was used as an internal loading control. (B) The results of the quantitative analyses are shown in the right panel. (**P* < 0.05, ***P*< 0.01, ****P* < 0.001).

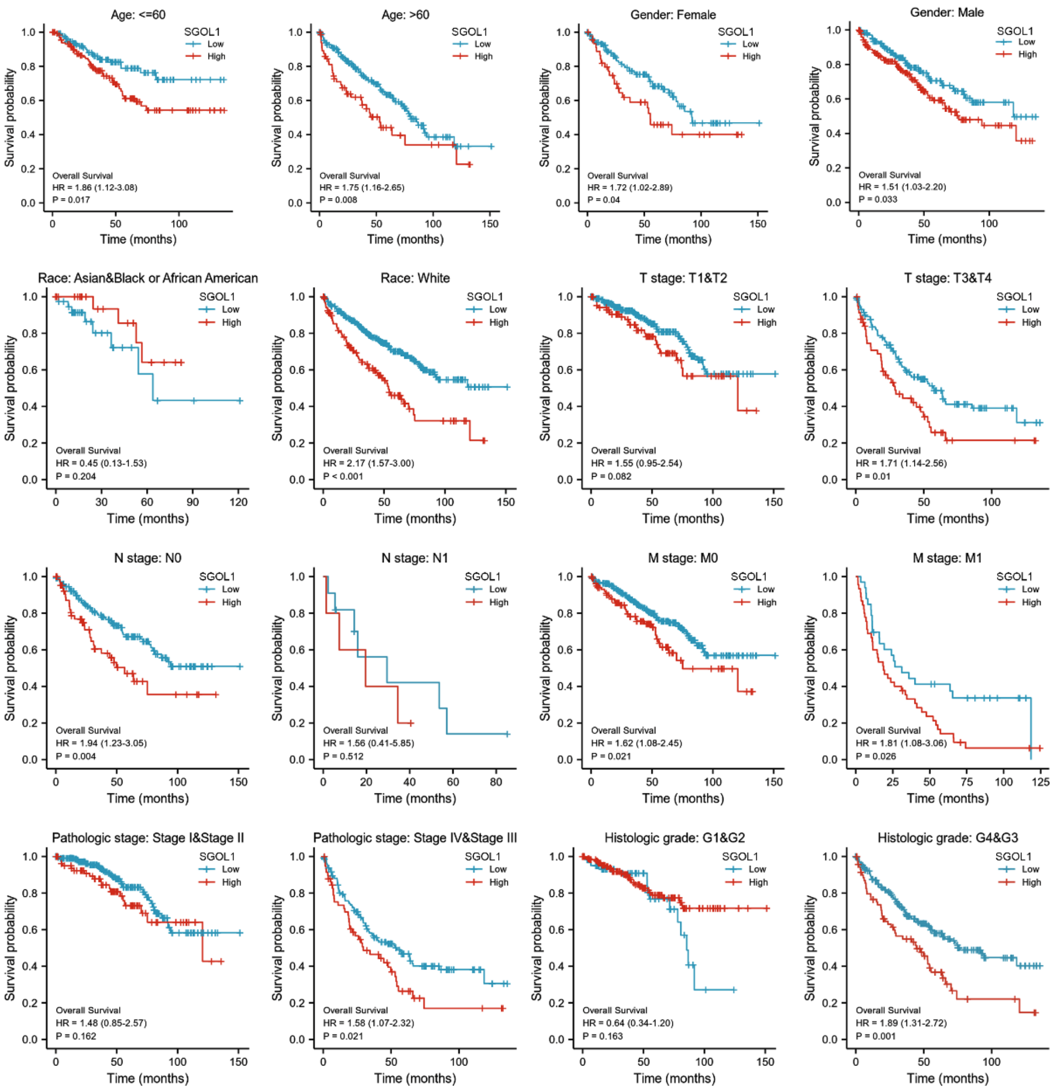

Supplementary Fig 3. Kaplan-Meier survival curves of SGOL1 in differential clinical-pathological features. Survival curves of OS between SGOL1 high and low subgroups among patients with age≤60, age＞60, Female, Male, Asian & Black or African American, White, T1&T2, T3&T4, N0, N1, M0, M1, stage I&II, stage III&IV, Grade 1&2, and Grade 3&4. Significance was set at P<0.05.

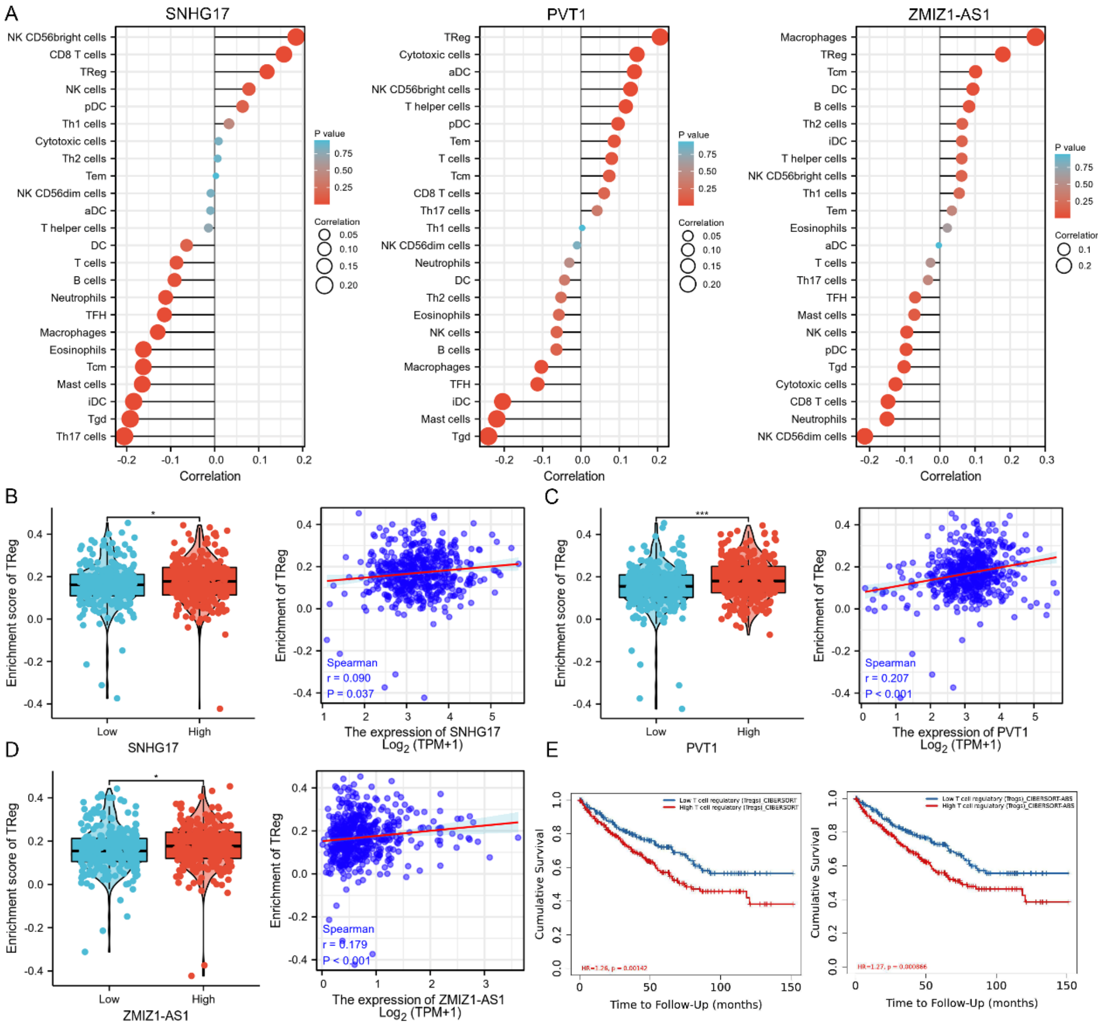

Supplementary Fig 4. The correlation analysis between the immune cell infiltration and lncRNAs (SNHG17, PVT1, and ZMIZ1-AS1) expression in KIRC. (A) The forest plot shows the correlations between the enrichment of 24 immune cells and lncRNAs (SNHG17, PVT1, and ZMIZ1-AS1) expression. (B-D) The enrichment of Treg cells was negatively correlated with lncRNAs (SNHG17, PVT1, and ZMIZ1-AS1) expression in KIRC. (E) The cumulative survival analysis of ccRCC patients in high-and-low Treg cell infiltration groups. (*$P < 0.05$, ***$P < 0.001$).
